# Supplementary material for: Explosive neural networks via higher-order interactions in curved statistical manifolds
Source: Nat Commun. 2025 Jul 24;16:6511. doi: 10.1038/s41467-025-61475-w (PMC12290047; doi:10.1038/s41467-025-61475-w)
Supplement: Supplementary file 1 — Supplementary Information [file 41467_2025_61475_MOESM1_ESM.pdf]

Explosive neural networks via higher-order interactions in curved statistical manifolds

Supplementary Information

Miguel Aguilera
BCAM – Basque Center for Applied Mathematics, Bilbao, Spain
IKERBASQUE, Basque Foundation for Science, Bilbao, Spain

Pablo A. Morales
Research Division, Araya Inc., Tokyo, Japan
Centre for Complexity Science, Imperial College London, London, UK

Fernando E. Rosas
Sussex AI and Sussex Centre for Consciousness Science,
Department of Informatics, University of Sussex, Brighton, UK
Department of Brain Science and Centre for Complexity Science, Imperial College London, London, UK
Center for Eudaimonia and Human Flourishing, University of Oxford, Oxford, UK
Principles of Intelligent Behavior in Biological and Social Systems (PIBBSS), Prague, Czech Republic

Hideaki Shimazaki
Graduate School of Informatics, Kyoto University, Kyoto, Japan
Center for Human Nature, Artificial Intelligence,
and Neuroscience (CHAIN), Hokkaido University, Sapporo, Japan

Supplementary Note 1: Maximum Rényi entropy and information geometry

The maximum entropy principle (MEP) is a framework for building parsimonious models consistent with observations, being particularly well-suited for the statistical description of systems in contexts of incomplete knowledge [38, 39]. The MEP uses entropy as a fundamental tool to quantify the degree of structure present in a given model. Accordingly, the MEP suggest to adopt the model with the maximal entropy — i.e. the least amount of structure — that is consistent with selected features of the data (for example, their first- and second-order statistics), following the idea that no additional regularities should be introduced beyond the ones specified by those.

Maximum entropy models are particularly well-suited for the study of neural systems. By abstracting neurons into binary variables x_i representing the presence or absence of action potentials, the MEP provides a powerful approach to model collective neural activity. In this approach, the Ising model emerges from the maximisation of Shannon entropy under constraints on activity rates of individual neurons and pairwise correlations:

$$p^{(2)} = \arg\max_{q(\boldsymbol{x})} H(\boldsymbol{x}) \quad \text{s.t.} \quad \begin{cases} \langle x_i \rangle & = \eta_i, \\ \langle x_i x_j \rangle & = \eta_{ij}, \end{cases}$$

where $H(\boldsymbol{x})$ denotes the Shannon entropy of $\boldsymbol{x} = \{x_1, \ldots, x_n\}$ under distribution $q(\boldsymbol{x})$. It can be shown that

$$p^{(2)}(\boldsymbol{x}) = \frac{1}{Z} \exp\left(\sum_i \theta_i x_i + \sum_{i<j} \theta_{ij} x_i x_j \right), \tag{S1.1}$$

with Z being a normalising constant. Hence, this model encapsulates observed information up to second-order statistics, represented in how θ_i, θ_{ij} depend on the constraints η_i, η_{ij}. Furthermore, the dynamics of the Ising model can be investigated via exact solutions, approximations (encompassing mean-field and Bethe approximations), and simulations, thereby providing a rich set of insights and analytical tools. The Ising model has been instrumental in the development of recurrent neural networks, leading to Hopfield networks and Boltzmann machines.

What if the observations that one is to model require us to consider statistics beyond pairwise interactions? Following the same principle, one can construct models with third- and higher-order interactions [29] resulting in distributions of the following type:

$$p^{(k)}(\boldsymbol{x}) = \frac{1}{Z} \exp\left(\sum_{\substack{\boldsymbol{I} \subseteq \{1,\ldots,n\} \\ |\boldsymbol{I}| \leq k}} \theta_{\boldsymbol{I}} \prod_{i \in \boldsymbol{I}} x_i \right) \tag{S1.2}$$

with the summation going over all subsets of k or less variables. Above, the argument within the exponential is an energy function $E_k(\boldsymbol{x})$, with the index k highlighting the highest order of interactions considered. It is important to notice that the number of terms in the Hamiltonian grows exponentially with k, making it unfeasible in practice to construct models including high orders $k \gg 1$.

1. Capturing high-order interactions via non-Shannon entropies

While traditional formulations of the MEP are based on Shannon's entropy [40], more recent work has expanded it to include other entropy functionals, including the entropies of Tsallis [41] and Rényi [42]. Here we argue that some high-order interdependencies can be efficiently captured by the deformed exponential family (2), which arises as a solution to the problem of maximising non-Shannon entropies — as explained below.

By starting from a conventional MEP model with few degrees of freedom tuned to account for low-order interactions, one can enhance its capability to account for higher-order interdependencies by the inclusion of a deformation parameter, defined as an extension of the Rényi's index (or Tsallis's q or Amari's α), with clear geometrical interpretation, i.e. the scalar curvature of the manifold [42]. Concretely, let's consider the Rényi entropy with parameter $\gamma \geq -1$, given by

$$H_\gamma = -\frac{1}{\gamma} \ln \sum_{\boldsymbol{x}} p(\boldsymbol{x})^{1+\gamma}. \tag{S1.3}$$

This definition adopts the shifted indexing convention introduced in Ref. [43], thereby referring to $\gamma = \alpha - 1$ as the order of Rényi's entropy, with $\alpha \geq 0$ corresponding to the order in the standard definition. Rényi entropy recovers the standard Shannon entropy at the limit $\gamma \to 0$. The maximisation of the Rényi entropy can be performed by extremisation of the Lagrangian:

$$\mathcal{L} = -\frac{1}{\gamma} \ln \sum_{\boldsymbol{x}} p(\boldsymbol{x})^{1+\gamma} + \theta_0 \left(\sum_{\boldsymbol{x}} p(\boldsymbol{x}) - 1 \right) + \theta_0 \gamma \beta \sum_{i=1}^{L} \theta_i \left(\sum_{\boldsymbol{x}} p(\boldsymbol{x}) f_i(\boldsymbol{x}) - c_i \right), \tag{S1.4}$$

which also consider constraints $\sum_{\boldsymbol{x}} p(\boldsymbol{x}) = 1$ and $\sum_{\boldsymbol{x}} p(\boldsymbol{x}) f_i(\boldsymbol{x}) = c_i$ with $i = 1, \ldots, L$, whereas the first ensures $p(\boldsymbol{x})$ to be a probability mass function and the second fixes the average of $f_i(\boldsymbol{x})$ on a desired value c_i. Note that the coefficient β is introduced to keep γ dimensionless, corresponding to the inverse temperature in statistical physics. This results in the maximum entropy condition

$$0 = \frac{\delta \mathcal{L}}{\delta p(\boldsymbol{x})} = -\frac{1}{\gamma} \frac{(1+\gamma) p(\boldsymbol{x})^\gamma}{\sum_{\boldsymbol{x}} p(\boldsymbol{x})^{1+\gamma}} + \theta_0 + \theta_0 \gamma \beta \sum_a \theta_a f_a(\boldsymbol{x}). \tag{S1.5}$$

The family of probability distributions meeting the above condition is known as the *deformed exponential family*, which is given by

$$p_\gamma(\boldsymbol{x}) = \exp(-\varphi_\gamma) \left[1 + \gamma \beta \sum_a \theta_a f_a(\boldsymbol{x}) \right]_+^{1/\gamma} \tag{S1.6}$$

where φ_γ is a normalising constant

$$\varphi_\gamma = \ln \sum_{\boldsymbol{x}} \left[1 + \gamma \beta \sum_a \theta_a f_a(\boldsymbol{x}) \right]_+^{1/\gamma}. \tag{S1.7}$$

Above, we use the square bracket $[\cdot]_+$ operator to set negative values to zero, so that $[x]_+ = \max\{0, x\}$. In the next sections, to solve the steepest descent step of mean field calculations, we will assume that the content of the $[\cdot]_+$ operator is always possible. This assumption is reasonable under an adequate normalisation of γ.

Importantly, Rényi's entropy is closely related to Tsallis' entropy

$$H_\gamma^{(\mathrm{Ts})} = -\frac{1}{\gamma} \left(1 - \sum_{\boldsymbol{x}} p(\boldsymbol{x})^{1+\gamma} \right). \tag{S1.8}$$

It can be shown that the Tsallis and Rényi's entropies can be deformed into one another by a monotonically increasing function. This fact brings both divergences, from the geometrical perspective, to the same equivalence class generating the same geometry, see Ref. [42]. In particular, by maximising Tsallis entropy, one recovers the same deformed exponential family, p_γ, using $q = 1 - \gamma$ [44].

We also note that maximising Rényi's entropy with constraints from the expectation given by the escort distribution leads to a similar distribution, but with the exponent replaced by $-1/\gamma$ (see Theorem 3.15 in [45]).

Supplementary Note 2: Glauber rule

Glauber dynamics is a Markov Chain Monte Carlo algorithm that is popular for simulating neural activity according to Hopfield networks and Ising models. In this method, one samples the activity of each neuron conditioned on the activity of other neurons according to the following conditional distribution:

$$p_\gamma(x_k|\boldsymbol{x}_{\setminus k}) = \frac{p_\gamma(x_k, \boldsymbol{x}_{\setminus k})}{p_\gamma(\boldsymbol{x}_{\setminus k})} = \frac{p_\gamma(x_k, \boldsymbol{x}_{\setminus k})}{p_\gamma(x_k, \boldsymbol{x}_{\setminus k}) + p_\gamma(-x_k, \boldsymbol{x}_{\setminus k})} = \frac{1}{1 + \frac{p_\gamma(-x_k, \boldsymbol{x}_{\setminus k})}{p_\gamma(x_k, \boldsymbol{x}_{\setminus k})}}, \tag{S2.1}$$

where $\boldsymbol{x}_{\setminus k}$ denotes the state of all neurons except the k-th one. This sampling procedure is carried out for all neurons in an iterative manner.

Let us construct Glauber dynamics for a curved neural network. The deformed exponential family distribution states that the distribution of $\boldsymbol{x}$ is given by

$$p_\gamma(\boldsymbol{x}) = \exp\left(-\varphi_\gamma\right) \left[1 - \gamma\beta E(\boldsymbol{x})\right]_+^{1/\gamma}, \tag{S2.2}$$

where the energy function $E(\boldsymbol{x})$ is given by

$$E(\boldsymbol{x}) = -\sum_i H_i x_i - \frac{1}{2N}\sum_{i,j} J_{ij} x_i x_j \tag{S2.3}$$

with $J_{ii} = 0$ and $J_{ij} = J_{ji}$. The deformed exponential family distribution can be rewritten as

$$p_\gamma(-x_k, \boldsymbol{x}_{\setminus k}) = \exp\left(-\varphi\right) \left[1 - \gamma\beta\left(E(\boldsymbol{x}) + 2x_k h_k\right)\right]_+^{1/\gamma} \tag{S2.4}$$

with $h_k = H_k + \frac{1}{N}\sum_j J_{kj} x_j$. Under the assumption of $1 - \gamma\beta E(\boldsymbol{x}) > 0$ (and the same for the state resulting from flipping the k-th spin), a direct derivation shows that

$$\begin{aligned} p_\gamma(x_k|\boldsymbol{x}_{\setminus k}) &= \left(1 + \left(\frac{1 - \gamma\beta\left(E(\boldsymbol{x}) + 2x_k h_k\right)}{1 - \gamma\beta E(\boldsymbol{x})}\right)^{1/\gamma}\right)^{-1} \\ &= \left(1 + \left(1 - \gamma 2\beta' x_k h_k\right)^{1/\gamma}\right)^{-1} \\ &= \left(1 + \exp_\gamma\left(-2\beta' x_k h_k\right)\right)^{-1} \qquad \text{(S2.5)} \\ \beta' &= \frac{\beta}{1 - \gamma\beta E(\boldsymbol{x})}. \qquad \text{(S2.6)} \end{aligned}$$

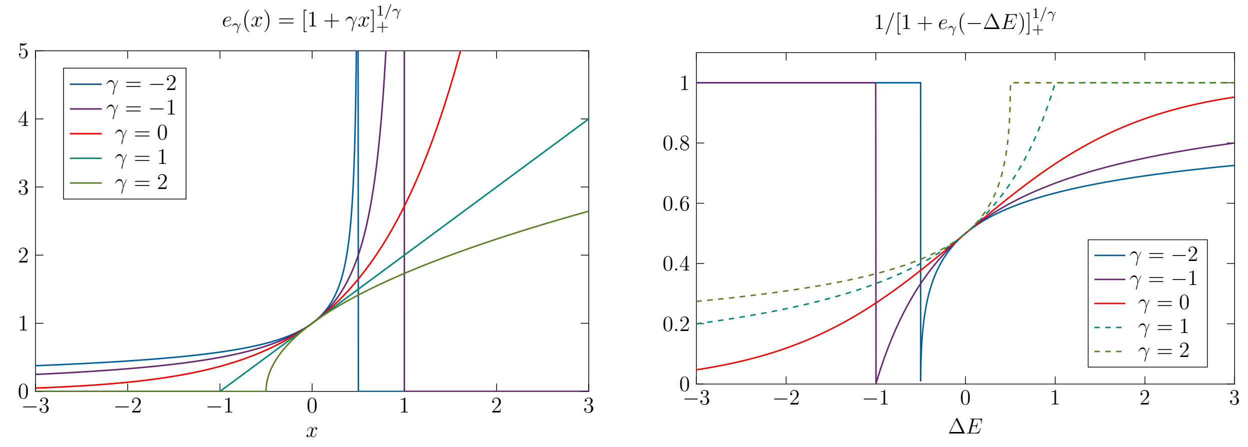

FIG. S1. **(Left)** The deformed exponential functions, $e_\gamma(x) = [1+\gamma x]_+^{\frac{1}{\gamma}}$. **(Right)** The activation function of a neuron as a function of input ΔE.

where $\exp_\gamma(\cdot)$ stands for the deformed exponential. Note that these equations recover the classical Glauber rule for Ising models at $\gamma = 0$. Fig. S1 shows the deformed exponential function and the activation function $p_\gamma(x_k = 1|\boldsymbol{x}_{\setminus k})$ as a function of input $\Delta E = 2\beta' h_k$, representing the deformed nonlinearity of a neuron. Note that to have a smooth activation function, the input must satisfy $1 + \gamma\Delta E > 0$, resulting in $\Delta E > -1/\gamma$ if $\gamma > 0$ and $\Delta E < -1/\gamma$ if $\gamma < 0$. For implementing the sampling strategy, the selection of neurons can be sequential, using random permutations, or using probabilistic methods (according to non-zero probabilities assigned to each neuron).

In the case of large systems in which $E(\boldsymbol{x})$ is extensive, then a normalisation of the curvature parameter in the form $\gamma' = \frac{\gamma}{N}$ is required. This makes the value of $\gamma x_k h_k$ tend to zero as $N \to \infty$. In this case, calculating the limit of $\exp_\gamma(-2\beta' x_k h_k)$ as $\gamma \to 0$, one finds that

$$p_\gamma(x_k|\boldsymbol{x}_{\setminus k}) = \left(1 + \exp\left(-2\beta' x_k h_k\right)\right)^{-1} = \frac{\exp\left(\beta' x_k h_k\right)}{2\cosh\left(\beta' h_k\right)}, \tag{S2.7}$$

with effective temperature β' given by

$$\beta' = \frac{\beta}{1 - \gamma' \frac{1}{N} E(\boldsymbol{x})}. \tag{S2.8}$$

Supplementary Note 3: The mean-field theory of curved neural network

1. Derivation of general mean-field solution

In this section, we study a curved neural network composed of N neurons that stores M patterns $\boldsymbol{\xi}^a = (\xi_1^a, \ldots, \xi_N^a)$, as described by the deformed exponential family distribution given by

$$p_\gamma(\boldsymbol{x}) = \exp\left(-\varphi_\gamma\right)\left[1+\gamma\beta\left(H\sum_{a,i}\xi_i^a x_i + \frac{J}{N}\sum_{a,i<j} x_i\xi_i^a\xi_j^a x_j\right)\right]_+^{1/\gamma}, \tag{S3.1}$$

where φ_γ is the normalising potential and γ is the deformation parameter. In the following sections, we assume that parameters are scaled so that the content of the brackets $[]_+$ is always positive to avoid non-differentiable values.

We start the analysis by computing the value of $\exp(\varphi)$ in the large N limit, which can be done employing a delta integral substituting the value of $\frac{1}{N}\sum_i x_i$:

$$\begin{aligned}\exp\left(\varphi_\gamma\right) &= \sum_{\boldsymbol{x}}\left[1+\gamma\beta\left(H\sum_{a,i}\xi_i^a x_i + \frac{J}{N}\sum_{a,i<j} x_i\xi_i^a\xi_j^a x_j\right)\right]_+^{1/\gamma} \\ &= \sum_{\boldsymbol{x}}\exp\left(\frac{1}{\gamma}\ln\left(1+\gamma\beta\left(H\sum_{a,i}\xi_i^a x_i + \sum_a\frac{J}{2N}\left(\left(\sum_i \xi_i^a x_i\right)^2 - N\right)\right)\right)\right),\end{aligned} \tag{S3.2}$$

where the second equality uses $(\sum_i x_i)^2 - N = 2\sum_{i<j}x_i x_j$. Additionally, by replacing $\frac{1}{N}\sum_i \xi_i^a x_i$ by a Dirac delta function under an integral, and then using the delta function's integral form $\delta(x-a) = \frac{1}{2\pi}\int_{-\infty}^{\infty} e^{-\mathrm{i}\zeta(x-a)}\,d\zeta$, the expression above can be re-written as

$$\begin{aligned}\exp\left(\varphi_\gamma\right) &= \sum_{\boldsymbol{x}}\int d\boldsymbol{m}\prod_a\delta\left(m_a - \frac{1}{N}\sum_i\xi_i^a x_i\right)\exp\left(\frac{1}{\gamma}\ln\left(1+\gamma N\beta\sum_a\left(Hm_a + \frac{J}{2}\left(m_a^2 - \frac{1}{N}\right)\right)\right)\right) \\ &= \frac{1}{(2\pi)^M}\int d\boldsymbol{m}d\hat{\boldsymbol{m}}\sum_{\boldsymbol{x}}\exp\left(\frac{1}{\gamma}\ln\left(1+\gamma N\beta\sum_a\left(Hm_a + \frac{J}{2}\left(m_a^2-\frac{1}{N}\right)\right)\right) - \sum_a \mathrm{i}\hat{m}_a\left(m_a - \frac{1}{N}\sum_i\xi_i^a x_i\right)\right).\end{aligned} \tag{S3.3}$$

Let us now introduce a scaling rule for the deformation parameter γ given by

$$\gamma = \frac{\gamma'}{\beta N}, \tag{S3.4}$$

where γ' is a constant independent of N, which is motivated by subsequent results for the mean-field solution that suggest this relationship between γ and N in order to maintain scale-invariant properties. Then, the potential φ_γ can be expressed in terms of γ' as

$$\begin{aligned}\exp\left(\varphi_\gamma\right) = \frac{1}{(2\pi)^M}\int d\boldsymbol{m}d\hat{\boldsymbol{m}}\exp\Bigg(&\frac{N\beta}{\gamma'}\ln\left(1+\gamma'\sum_a\left(Hm_a + \frac{J}{2}\left(m_a^2 - \frac{1}{N}\right)\right)\right) \\ &-\sum_a \mathrm{i}\hat{m}_a m_a + \sum_i\ln\left(2\cosh\left(\frac{1}{N}\sum_a\xi_i^a\mathrm{i}\hat{m}_a\right)\right)\Bigg).\end{aligned} \tag{S3.5}$$

Under this condition, the exponent in the equation above goes to infinity as $N\to\infty$. In this limit, the integral can be evaluated by the method of the steepest descent (a.k.a. the saddle-point method), yielding

$$\begin{aligned}\exp\left(\varphi_\gamma\right) = \exp\Bigg\{&\frac{N\beta}{\gamma'}\ln\left(1+\gamma'\sum_a\left(Hm_a + \frac{J}{2}\left(m_a^2-\frac{1}{N}\right)\right)\right) \\ &-\sum_a\mathrm{i}\hat{m}_a m_a + \sum_i\ln\left(2\cosh\left(\frac{1}{N}\sum_a\xi_i^a\mathrm{i}\hat{m}_a\right)\right)\Bigg\}\end{aligned} \tag{S3.6}$$

where the content of the brackets set to the values of $\boldsymbol{m}, \hat{\boldsymbol{m}}$ that extremises (i.e. maximise or minimise) its content. To obtain their values we find the values that make the derivative of the expression inside the brackets equal to zero. By differentiating the exponent by $\hat{m}_a$, we find the saddle point must satisfy

$$m_a = \frac{1}{N}\sum_i \xi_i^a \tanh\left(\frac{1}{N}\sum_b \xi_i^b \mathrm{i}\hat{m}_b\right). \tag{S3.7}$$

Similarly, differentiating by m_a yields

$$\mathrm{i}\hat{m}_a = \beta' N(H + Jm_a), \tag{S3.8}$$

where we introduced the effective inverse temperature β':

$$\beta' = \frac{\beta}{1 + \gamma' \sum_b \left(Hm_b + \frac{J}{2}m_b^2\right)}. \tag{S3.9}$$

From these equations, we find the mean-field solution in the limit of large N:

$$m_a = \frac{1}{N}\sum_i \xi_i^a \tanh\left(\beta' \sum_b \xi_i^b (H + Jm_b)\right), \tag{S3.10}$$

which recovers the classical mean field solution at $\gamma' = 0$. This solution confirms that γ has to be scaled by the system size to maintain the scale-invariant properties.

The normalising potential in the large N limit is obtained as

$$\varphi_\gamma = \frac{\beta N}{\gamma'} \ln \frac{\beta'}{\beta} - \sum_a m_a \beta' N(H + Jm_a) + \sum_i \ln\left(2\cosh\left(\beta' \sum_a \xi_i^a (H + Jm_a)\right)\right). \tag{S3.11}$$

2. A single pattern: explosive phase transitions

When embedded memory contains only a single pattern ($M = 1$), the equations above result in

$$\varphi_\gamma = \frac{\beta N}{\gamma'} \ln \frac{\beta'}{\beta} - N\beta'(H + Jm)m + N \ln\left(2\cosh\left(\beta'(H + Jm)\right)\right), \tag{S3.12}$$

with

$$m = \tanh\left(\beta'(H + Jm)\right), \tag{S3.13}$$

$$\beta' = \frac{\beta}{1 + \gamma'(Hm + \frac{J}{2}m^2)}. \tag{S3.14}$$

Under the limit of small γ given by the scaling (S3.4), the derivative of the normalisation potential φ_γ w.r.t. H yields the corresponding expected value, similarly to the exponential family distribution. Then, $\gamma' = 0$ yields the classical result. This can be verified by

$$\begin{aligned}\frac{\partial \varphi_\gamma}{\partial H} &= -\frac{\beta N}{\gamma'}\frac{\partial \beta'}{\partial H} - N\beta'(H + Jm)\frac{\partial m}{\partial H} - N\frac{\partial \beta'(H + Jm)}{\partial H}m + N\frac{\partial \beta'(H + Jm)}{\partial H}m \\ &= -\frac{\beta N}{\gamma'}\frac{\partial \beta'}{\partial H} - N\beta'(H + Jm)\frac{\partial m}{\partial H},\end{aligned} \tag{S3.15}$$

where

$$\frac{\partial \beta'}{\partial H} = \frac{-\beta\gamma'\left(m + \frac{\partial m}{\partial H}(H + Jm)\right)}{\left(1 + \gamma'(Hm + \frac{J}{2}m^2)\right)^2} = -\beta^{-1}\beta'^2\gamma'\left(m + \frac{\partial m}{\partial H}(H + Jm)\right), \tag{S3.16}$$

leading to

$$\frac{\partial \varphi_\gamma}{\partial H} = N\beta'\left(m + \frac{\partial m}{\partial H}(H + Jm)\right) - N\beta'(H + Jm)\frac{\partial m}{\partial H} \tag{S3.17}$$

$$= N\beta' m. \tag{S3.18}$$

The result recovers the classical relation, $\frac{\partial \varphi_\gamma}{\partial H} = \beta N m$ for the case $\gamma' = 0$.

a. Behaviour at criticality

Now, we compute the critical exponents of the mean-field parameter for $H = 0$. In the thermodynamic limit with $\gamma = \gamma'/(\beta N)$, one finds that

$$m = \tanh\left(\frac{\beta J m}{1 + \gamma' \frac{J}{2} m^2}\right). \tag{S3.19}$$

Since $\tanh\left(\frac{am}{1+bm}\right) = am - (a^3/3 + ab)m^3 + O(m^4)$, by expanding the r.h.s. around $m = 0$ up to the third order, one can find that

$$m = \beta J m - \frac{1}{6}(\beta J)\left(2(\beta J)^2 + 3J\gamma'\right) m^3 + \mathcal{O}(m^4), \tag{S3.20}$$

which yields a trivial solution at $m = 0$ and two non-trivial solutions given by

$$m_{\pm} = \pm\sqrt{\frac{\beta J - 1}{\frac{1}{6}\beta J\left(2(\beta J)^2 + 3J\gamma'\right)}}, \tag{S3.21}$$

which yields a mean-field universality class critical exponent 'beta' (not to be confused with the inverse temperature) of $\frac{1}{2}$.

The magnetic susceptibility, $\chi := \frac{\partial m}{\partial H}$, of the deformed Ising model can be calculated using (S3.14). Hence, we have

$$\frac{\partial m}{\partial H} = (1 - m^2)\left(\beta'\left(1 + J\frac{\partial m}{\partial H}\right) + \frac{\partial \beta'}{\partial H}(H + Jm)\right). \tag{S3.22}$$

Using (S3.16), we obtain

$$\begin{aligned}\frac{\partial m}{\partial H} &= (1 - m^2)\left(\beta'\left(1 + J\frac{\partial m}{\partial H}\right) - \beta^{-1}\beta'^2\gamma'\left(m + \frac{\partial m}{\partial H}(H + Jm)\right)(H + Jm)\right)\\ &= (1 - m^2)\beta'\left(1 + J\frac{\partial m}{\partial H} - \frac{\beta'}{\beta}\gamma'\left(m + \frac{\partial m}{\partial H}(H + Jm)\right)(H + Jm)\right)\\ &= (1 - m^2)\beta'\left(1 - \frac{\beta'}{\beta}\gamma' m(H + Jm) + \frac{\partial m}{\partial H}\left(J - \frac{\beta'}{\beta}\gamma'(H + Jm)^2\right)\right).\end{aligned} \tag{S3.23}$$

Then, we obtain

$$\frac{\partial m}{\partial H} = \frac{(1 - m^2)\beta'\left(1 - \frac{\beta'}{\beta}\gamma' m(H + Jm)\right)}{1 - (1 - m^2)\beta'\left(J - \frac{\beta'}{\beta}\gamma'(H + Jm)^2\right)}. \tag{S3.24}$$

The susceptibility $\frac{dm}{dH}$ at $m = 0$ is

$$\frac{\partial m}{\partial H} = \frac{\beta}{1 - \beta\left(J - \frac{\beta'}{\beta}\gamma' H^2\right)} \tag{S3.25}$$

$$= \frac{-\beta\beta_c}{\beta - \beta_c}, \tag{S3.26}$$

where the critical inverse temperature is given by $\beta_c = 1/(J - \frac{\beta'}{\beta}\gamma' H^2)$. Thus, the susceptibility results in the universality class 'gamma' exponent of 1 (not to be confused with the deformation parameter) near the critical temperature. At $H = 0$, $\beta_c = 1/J$.

Furthermore, at $\gamma' = 0$, we recover

$$\frac{\partial m}{\partial H} = \frac{(1 - m^2)\beta}{1 - (1 - m^2)\beta J}. \tag{S3.27}$$

3. Two correlated patterns

Here we study an exemplary case in which two patterns are embedded in the deformed associative network, with $\frac{1}{N}\sum_i \xi_i^1\xi_i^2 = C$. Thus, the fraction of terms for which $\xi_i^1\xi_i^2 = \pm 1$ is equal to $\frac{1\pm C}{2}$. We seek solutions for (S3.10) and (11), which can be further simplified for the case of two correlated patterns. We note that the content of the tanh and cosh terms can only take two values for the two patterns $\xi_i^a = \pm 1$. In the case of the tanh terms

$$\begin{aligned}\frac{1}{N}\sum_i \xi_i^a \tanh\left(\beta' \sum_b \xi_i^b (H + Jm_b)\right) =& \frac{1}{N}\sum_i \tanh\left(\beta' \sum_b \xi_i^a \xi_i^b (H + Jm_b)\right) \\ =& \frac{1+C}{2}\tanh\left(\beta'(2H + J(H + J(m_1+m_2)))\right) + \frac{1-C}{2}\tanh\left(\beta' J(m_1 - m_2)\right).\end{aligned} \tag{S3.28}$$

Hence, by replacing terms, one can find that

$$m_1 = \frac{1+C}{2}\tanh\left(\beta'(2H + Jm_1 + Jm_2)\right) + \frac{1-C}{2}\tanh\left(\beta'(Jm_1 - Jm_2)\right), \tag{S3.29}$$

$$m_2 = \frac{1+C}{2}\tanh\left(\beta'(2H + Jm_1 + Jm_2)\right) - \frac{1-C}{2}\tanh\left(\beta'(Jm_1 - Jm_2)\right). \tag{S3.30}$$

The normalising potential then becomes

$$\begin{aligned}\varphi_\gamma =& \frac{\beta N}{\gamma'}\ln\frac{\beta'}{\beta} - \beta' N m_1 (H + Jm_1) - \beta' N m_2 (H + Jm_2) \\ &+ \frac{1+C}{2} N \ln\left(2\cosh\left(\beta'(2H + Jm_1 + Jm_2)\right)\right) + \frac{1-C}{2} N \ln\left(2\cosh\left(\beta'(Jm_1 - Jm_2)\right)\right).\end{aligned} \tag{S3.31}$$

Supplementary Note 4: Dynamical mean-field theory

Let us now describe the statistics of temporal trajectories of the system. For this, let's consider the trajectory $\boldsymbol{x}_{0:T} = (\boldsymbol{x}_0, \ldots, \boldsymbol{x}_T)$, whose probability can be computed as

$$p_\gamma(\boldsymbol{x}_{0:T}) = \prod_t p_\gamma(\boldsymbol{x}_t|\boldsymbol{x}_{t-1}), \tag{S4.1}$$

where the probability of the transition between $\boldsymbol{x}_{t-1}$ and $\boldsymbol{x}_t$ can be expressed as

$$p_\gamma(\boldsymbol{x}_t|\boldsymbol{x}_{t-1}) = \frac{1}{N}\sum_i p_\gamma(x_{i,t}|\boldsymbol{x}_{t-1}) \prod_{j:j\neq i} \delta[x_{j,t}, x_{j,t-1}], \tag{S4.2}$$

using the Kronecker delta, $\delta[x,y]$. For large system sizes, individual transitions (see (S2.7)) can be expressed as

$$p_\gamma(x_{i,t}|\boldsymbol{x}_{t-1}) = \frac{\exp\left(\beta' x_{i,t} h_{i,t}\right)}{2\cosh\left(\beta' h_{i,t}\right)}, \tag{S4.3}$$

$$h_{i,t} = \sum_a \xi_i^a \left(H_a + \frac{1}{N} \sum_{j:j\neq i} \xi_j^a x_{j,t-1} \right), \tag{S4.4}$$

$$\beta'_t = \frac{\beta}{1 + \gamma' \left(\frac{1}{N}\sum_i x_{i,t-1} \sum_a \xi_i^a \left(H_a + \frac{1}{2N}\sum_{j:j\neq i} \xi_j^a x_{j,t-1} \right) \right)}. \tag{S4.5}$$

As before, the above derivation assumes that the content of the $[]_+$ operator in the definition of the deformed exponential family is non-negative.

Using the integral form of the Kronecker delta function, the above transition probability can be rewritten as

$$\begin{aligned} p_\gamma(\boldsymbol{x}_t|\boldsymbol{x}_{t-1}) &= \frac{1}{N}\sum_i p_\gamma(x_{i,t}|\boldsymbol{x}_{t-1}) \prod_{j:j\neq i} \delta[1, x_{j,t}x_{j,t-1}] \\ &= \frac{1}{N(2\pi)^{N-1}} \sum_i \int_{\mathbf{0}}^{\mathbf{2\pi}} d\boldsymbol{\phi}_t \exp\left(\beta'_t x_{i,t} h_{i,t} - \ln\left(2\cosh\left(\beta'_t h_{i,t}\right)\right) + \sum_{j:j\neq i} \mathrm{i}\phi_{j,t}(1 - x_{j,t}x_{j,t-1}) \right). \end{aligned} \tag{S4.6}$$

Let $k_t \in (1, \ldots, N)$ be a uniform independent random variable. Namely, at each time step t, the index k_t is drawn independently and uniformly from the set $\{1, 2, \ldots, N\}$. Then, the sequence $\{k_t\}_{t=1}^T$ constitutes auxiliary variables to keep track of which spin is being updated at each time step. Using k_t, the average over the spin in the equation above can be replaced by an average over the uniform k_t: $\frac{1}{N}\sum_{i=1}^N \rightarrow \sum_{k_t=1}^N \frac{1}{N}$. Using this, the probability of the trajectory $\boldsymbol{x}_{0:t}$ can be rewritten as

$$\begin{aligned} p_\gamma(\boldsymbol{x}_{0:T}) = &\frac{1}{N^T (2\pi)^{(N-1)T}} \sum_{\boldsymbol{k}} \int_{\mathbf{0}}^{\mathbf{2\pi}} d\boldsymbol{\phi} \exp\Bigg(\sum_{i,t} \Bigg(\left(\beta'_t x_{i,t} h_{i,t} - \ln\left(2\cosh\left(\beta'_t h_{i,t}\right)\right)\right) \delta[i, k_t] \\ &\qquad - \sum_{j:j\neq i} \mathrm{i}\phi_{j,t}(1 - x_{j,t}x_{j,t-1})(1 - \delta[j, k_t]) \Bigg)\Bigg) \\ = &\frac{1}{N^T (2\pi)^{(N-1)T}} \sum_{\boldsymbol{k}} \int_{\mathbf{0}}^{\mathbf{2\pi}} d\boldsymbol{\phi} \exp\Bigg(\sum_{i,t} \Bigg(\left(\beta'_t x_{i,t} h_{i,t} - \ln\left(2\cosh\left(\beta'_t h_{i,t}\right)\right)\right) \delta[i, k_t] \\ &\qquad - (N-1)\mathrm{i}\phi_{i,t}(1 - x_{i,t}x_{i,t-1})(1 - \delta[i, k_t]) \Bigg)\Bigg) \\ = &\lim_{\lambda\to\infty} \frac{1}{N^T} \sum_{\boldsymbol{k}} \exp\left(\sum_{i,t} \left(\left(\beta'_t x_{i,t} h_{i,t} - \ln\left(2\cosh\left(\beta'_t h_{i,t}\right)\right)\right) \delta[i, k_t] - \lambda(N-1)(1 - x_{i,t}x_{i,t-1})(1 - \delta[i, k_t]) \right)\right), \end{aligned} \tag{S4.7}$$

where the second equality switches i, j indices and the third equality can be justified by the Γ-convergence of the log probability functionals

$$\limsup_{\lambda\to\infty} \left(-\lambda C(1-xy)(1-\delta)\right) \geq \ln \frac{1}{2\pi}\int_0^{2\pi} d\phi \, \exp\left(-i\phi\, C(1-xy)(1-\delta)\right), \tag{S4.8}$$

which substitutes the oscillatory delta integral by a soft exponential penalty with a large parameter λ, ensuring that the minimizers of the soft-penalized functional converge to those of the original constrained system.

As (with $\delta \in [0,1]$) we have $\lim_{\lambda\to\infty} \ln\cosh(k \pm \lambda(1-\delta)) = \lim_{\lambda\to\infty}(\ln\cosh(k)\delta + \lambda(1-\delta))$, we can simplify the equation above as follows:

$$p_\gamma(\boldsymbol{x}_{0:T}) = \lim_{\lambda\to\infty} \frac{1}{N^T}\sum_{\boldsymbol{k}} \exp\left(\sum_{i,t}\left(\beta'_t x_{i,t} h_{i,t}^{\lambda,k_t} - \ln\left(2\cosh\left(\beta'_t h_{i,t}^{\lambda,k_t}\right)\right)\right)\right), \tag{S4.9}$$

$$h_{i,t}^{\lambda,k_t} = \sum_a \xi_i^a \left(H_a + \frac{1}{N}\sum_{j:j\neq i}\xi_j^a x_{j,t-1}\right) + \beta_t'^{-1}\lambda(N-1)x_{i,t-1}(1-\delta[i,k_t]). \tag{S4.10}$$

This operation absorbs the second term in (S4.7) related to "the spin i not chosen" ($\delta[i,k_t]=0$) into the effective field $h_{i,t}^{\lambda,k_t}$, ensuring the strong coupling of the current state $x_{i,t}$ with the previous state $x_{i,t-1}$ using large λ. One can verify that it recovers (S4.7) by noting that if $\delta[i,k_t]=1$ (spin i chosen), then $h_{i,t}^{\lambda,k_t} = h_{i,t}$, recovering the first term in the exponent of (S4.7), and that if $\delta[i,k_t]=1$ (spin i not chosen), we have $h_{i,t}^{\lambda,k_t} \sim \beta_t'^{-1}\lambda(N-1)x_{i,t-1}$ for large λ, which yields

$$\beta'_t x_{i,t} h_{i,t}^{\lambda,k_t} - \ln\left(2\cosh\left(\beta'_t h_{i,t}^{\lambda,k_t}\right)\right) \sim \lambda(N-1)x_{i,t}x_{i,t-1} - |\lambda(N-1)x_{i,t-1}| = \lambda(N-1)(1-x_i x_{i,t-1}), \tag{S4.11}$$

recovering the second term in (S4.7).

In equilibrium systems, the partition function retrieves their statistical moments. A nonequilibrium equivalent function is a generating functional or dynamical partition function [59]. Let us now define the generating functional

$$Z(\boldsymbol{g}) = \sum_{\boldsymbol{x}} \exp\left(\sum_{i,t} g_{i,t}x_{i,t}\right) p_\gamma(\boldsymbol{x}_{0:T}), \tag{S4.12}$$

such that the following relationship is satisfied:

$$\frac{dZ(\mathbf{0})}{dg_{i,t}} = \langle x_{i,t}\rangle\,. \tag{S4.13}$$

Then, one can find an analytical expression for the functional by introducing delta integrals. Defining

$$\tilde{h}_{i,t}^{\lambda,k_t} = \sum_a \xi_i^a (H_a + m_{a,t-1}) + \beta_t'^{-1}(N-1)\lambda x_{i,t-1}(1-\delta[i,k_t]), \tag{S4.14}$$

$$\tilde{\beta}'_t = \frac{\beta}{1+\gamma'\sum_a\left(H_a m_{a,t-1} + \frac{1}{2}m_{a,t-1}^2\right)}, \tag{S4.15}$$

we obtain

$$\begin{aligned}
Z(\boldsymbol{g}) &= \lim_{\lambda\to\infty}\frac{1}{N^T}\sum_{\boldsymbol{x}}\sum_{\boldsymbol{k}}\exp\left(\sum_{i,t}\left(x_{i,t}(g_{i,t}+\tilde{\beta}'_t h_{i,t}^{\lambda,k_t}) - \ln\left(2\cosh\left(\tilde{\beta}'_t h_{i,t}^{\lambda,k_t}\right)\right)\right)\right) \\
&= \lim_{\lambda\to\infty}\frac{1}{N^T(2\pi)^{MT}}\sum_{\boldsymbol{x}}\int d\boldsymbol{m}d\hat{\boldsymbol{m}}\sum_{\boldsymbol{k}}\exp\left(\sum_{i,t}\left(x_{i,t}(g_{i,t}+\tilde{\beta}'_t\tilde{h}_{i,t}^{\lambda,k_t}) - \ln\left(2\cosh\left(\tilde{\beta}'_t\tilde{h}_{i,t}^{\lambda,k_t}\right)\right)\right)\right. \\
&\quad \left. - \sum_{a,t} \mathrm{i}\hat{m}_{a,t}\left(m_{a,t} - \frac{1}{N}\sum_i \xi_i^a x_{i,t}\right)\right) \\
&= \lim_{\lambda\to\infty}\frac{1}{(2\pi)^{MT}}\int d\boldsymbol{m}d\hat{\boldsymbol{m}}\exp\left(-\sum_{a,t}\mathrm{i}\hat{m}_{a,t}m_{a,t} + \ln\left(\frac{1}{N^T}\sum_{\boldsymbol{x},\boldsymbol{k}} e^{L^{\boldsymbol{k}}}\right)\right),
\end{aligned} \tag{S4.16}$$

where $L^{\boldsymbol{k}}$ is given as

$$L^{\boldsymbol{k}} = \sum_{i,t} x_{i,t} \left(g_{i,t} + \tilde{\beta}'_t \tilde{h}^{\lambda,k_t}_{i,t} + \sum_a \xi_i^a \mathrm{i}\hat{m}_{a,t} \right) - \sum_{i,t} \ln \left(2\cosh \left(\tilde{\beta}'_t \tilde{h}^{\lambda,k_t}_{i,t} \right) \right). \tag{S4.17}$$

One can solve the mean-field equations via steepest descent, obtaining

$$m_{a,t} = \frac{1}{N} \sum_i \xi_i^a \left\langle x_{i,t} \right\rangle_{L^{\boldsymbol{k}}}, \tag{S4.18}$$

$$\mathrm{i}\hat{m}_{a,t} = \sum_a \xi_i^a \left\langle \left(x_{i,t} - \tanh \left(\tilde{\beta}'_t \tilde{h}^{\lambda,k_t}_{i,t} \right) \right) \right\rangle_{L^{\boldsymbol{k}}} \tag{S4.19}$$

with

$$\left\langle f(\boldsymbol{x}) \right\rangle_{L^{\boldsymbol{k}}} = \lim_{\lambda\to\infty} \frac{\frac{1}{N^T} \sum_{\boldsymbol{x},\boldsymbol{k}} f(\boldsymbol{x}) e^{L^{\boldsymbol{k}}}}{\frac{1}{N^T} \sum_{\boldsymbol{x},\boldsymbol{k}} e^{L^{\boldsymbol{k}}}}. \tag{S4.20}$$

For $\boldsymbol{g} = \boldsymbol{0}$, we obtain $\mathrm{i}\hat{m}_{a,t} = 0$ and $\frac{1}{N^T} \sum_{\boldsymbol{x},\boldsymbol{k}} e^{L^{\boldsymbol{k}}} = 1$.

Let us also define as

$$\left\langle f(\boldsymbol{x}) \right\rangle_L = \frac{\sum_{\boldsymbol{x}} f(\boldsymbol{x}) e^L}{\sum_{\boldsymbol{x}} e^L}, \tag{S4.21}$$

where L is defined

$$L = \sum_{i,t} x_{i,t} \left(g_{i,t} + \tilde{\beta}'_t \tilde{h}_{i,t} + \sum_a \xi_i^a \mathrm{i}\hat{m}_{a,t} \right) - \sum_{i,t} \ln \left(2\cosh \left(\tilde{\beta}'_t \tilde{h}_{i,t} \right) \right), \tag{S4.22}$$

using

$$\tilde{h}_{i,t} = \sum_a \xi_i^a (H_a + m_{a,t-1}). \tag{S4.23}$$

One can relate L with $L^{\boldsymbol{k}}$ via the following equation,

$$\lim_{\lambda\to\infty} e^{L^{\boldsymbol{k}}} = e^L \prod_{i,t} (1 - \delta[i, k_t]) \delta[x_{i,t} x_{i,t-1}] \tag{S4.24}$$

forcing spins not to change sign when $\delta[i, k_{i,t}] = 0$. Note that, when $k_t \neq i$, spin $x_{i,t}$ takes the value of the spin at the previous step $x_{i,t-1}$.

The mean field behaviour is recovered for $\boldsymbol{g} = \boldsymbol{0}$, which we will assume from now on. This results in

$$\begin{aligned} \left\langle x_{i,t} \right\rangle_{L^{\boldsymbol{k}}} &= \lim_{\lambda\to\infty} \frac{1}{N^T} \sum_{\boldsymbol{x}_t \boldsymbol{k}} x_{i,t} e^{L^{\boldsymbol{k}}} \\ &= \lim_{\lambda\to\infty} \left(\frac{1}{N^{t-1}} \sum_{\boldsymbol{x}_t \boldsymbol{k}: k_t \neq i} x_{i,t} e^{L^{\boldsymbol{k}}} + \frac{1}{N^{t-1}} \sum_{\boldsymbol{x}_t \boldsymbol{k}: k_t = i} x_{i,t} e^{L^{\boldsymbol{k}}} \right) \\ &= \left(1 - \frac{1}{N} \right) \left\langle x_{i,t-1} \right\rangle_{L^{\boldsymbol{k}}} + \frac{1}{N} \left\langle x_{i,t} \right\rangle_L \end{aligned} \tag{S4.25}$$

because of the effect of the δ function in (S4.14).

We also find that

$$\left\langle x_{i,t} \right\rangle_L = \tanh \left(\tilde{\beta}'_t \tilde{h}_{i,t} \right). \tag{S4.26}$$

This results in

$$m_{a,t} = \frac{1}{N} \sum_i \xi_i^a \left\langle x_{i,t} \right\rangle_{L^{\boldsymbol{k}}} = m_{a,t-1} \left(1 - \frac{1}{N} \right) + \frac{1}{N^2} \sum_i \xi_i^a \tanh \left(\tilde{\beta}'_t \tilde{h}_{i,t} \right). \tag{S4.27}$$

The expression above can be rearranged in the form similar to a differential equation

$$\frac{m_{a,t} - m_{a,t-1}}{N^{-1}} = = -m_{a,t-1} + \frac{1}{N}\sum_i \xi_i^a \tanh\left(\tilde{\beta}'_t \sum_b \xi_i^b (H_b + m_{b,t-1})\right). \tag{S4.28}$$

Under large N and for an adequate time re-scaling, this leads to the following differential equation:

$$\dot{m}_a = -m_a + \frac{1}{N}\sum_i \xi_i^a \tanh\left(\beta' \sum_b \xi_i^b (H_b + m_b)\right), \tag{S4.29}$$

$$\beta' = \frac{\beta}{1 + \gamma' \sum_a \left(H_a m_a + \frac{1}{2} m_a^2\right)}. \tag{S4.30}$$

Supplementary Note 5: Replica analysis near saturation

Here we analyse a curved neural network with an extensive number of patterns, $M = \alpha N$ in (5). The model involves integrals over a large number of variables, making the steepest descent method inapplicable. Instead, we adopt the approach reported in Ref. [62], and average the free energy over the distribution of patterns using the replica trick.

For $Z = \exp(\varphi_\gamma)$, the replica trick is applied as follows:

$$\langle\!\langle \varphi_\gamma \rangle\!\rangle = \langle\!\langle \ln Z \rangle\!\rangle = \lim_{n\to 0} \frac{1}{n}(\langle\!\langle Z^n \rangle\!\rangle - 1), \tag{S5.1}$$

which can be equivalently written as

$$\langle\!\langle \ln Z \rangle\!\rangle = \lim_{n\to 0} \frac{1}{n} \ln \langle\!\langle Z^n \rangle\!\rangle \tag{S5.2}$$

with $\langle\!\langle f(\boldsymbol{x}) \rangle\!\rangle = 2^{-MN} \sum_{\boldsymbol{\xi}} f(\boldsymbol{x})$ being the configurational average over different combinations of the systems' parameters.

1. General derivation

To calculate the encoding of patterns, we introduce $\{\boldsymbol{\xi}_a\}$ with $a = 1, ..., M$ where the first l patterns are given — called 'nominated' patterns — and we average over the $M - l$ rest. Again, assuming as in Supplementary Note 3 that the content of the $[\,]_+$ is positive, we calculate

$$\langle\!\langle Z^n \rangle\!\rangle = \frac{1}{2^{N(M-l)}} \sum_{\boldsymbol{\xi}^{a>l}} \sum_{\boldsymbol{x}} \exp\left(\frac{1}{\gamma} \sum_u \ln \left(1 + \gamma\beta \left(\sum_{b\le l} H_b \frac{1}{N} \sum_i x_i^u \xi_i^b + \frac{J}{N} \sum_a \sum_{i<j} x_i^u \xi_i^a \xi_j^a x_j^u \right)\right)\right). \tag{S5.3}$$

We want to compute the configurational average of a network with M memories with $N, M \to \infty$ and $M/N = \alpha$, introducing a pair of delta integrals

$$\begin{aligned}\langle\!\langle Z^n \rangle\!\rangle = \frac{1}{2^{N(M-l)}\,(2\pi)^{(l+1)n}} \int d\boldsymbol{m} d\hat{\boldsymbol{m}} d\boldsymbol{\mu} d\hat{\boldsymbol{\mu}} \sum_{\boldsymbol{\xi}^{a>l}} \sum_{\boldsymbol{x}} \exp\Bigg(&- \sum_{u,b\le l} \mathrm{i}\hat{m}_b^u \left(m_b^u - \frac{1}{N} \sum_i x_i^u \xi_i^b \right) \\ &- \sum_u \mathrm{i}\hat{\mu}_u \left(\mu_u - \frac{1}{2} \sum_{a>l} \left(\frac{1}{\sqrt{N}} \sum_i x_i^u \xi_i^a \right)^2 \right) \\ &\frac{1}{\gamma} \sum_u \ln \left(1 + \gamma\beta \left(\sum_{b\le l} \left(N H_b m_b^u + \frac{JN}{2} (m_b^u)^2 \right) + J\mu_u - N\frac{J\alpha}{2} \right)\right)\Bigg),\end{aligned} \tag{S5.4}$$

where the $N\frac{J\alpha}{2}$ comes from substracting the diagonal. This leads to

$$\begin{aligned}\langle\!\langle Z^n \rangle\!\rangle = \frac{1}{(2\pi)^{(l+1)n}} \int d\boldsymbol{m} d\hat{\boldsymbol{m}} d\boldsymbol{\mu} d\hat{\boldsymbol{\mu}} \sum_{\boldsymbol{x}} \exp\Bigg(&\frac{1}{\gamma} \sum_u \ln \left(1 + \gamma\beta \left(\sum_{b\le l} \left(N H_b m_b^u + \frac{JN}{2} (m_b^u)^2 \right) + J\mu_u - N\frac{J\alpha}{2} \right)\right) \\ &- \sum_{u,b\le l} \mathrm{i}\hat{m}_b^u \left(m_b^u - \frac{1}{N} \sum_i x_i^u \xi_i^b \right) - \sum_u \mathrm{i}\hat{\mu}_u \mu_u + \ln \left(\frac{1}{2^{N(M-l)}} \sum_{\boldsymbol{\xi}^{a>l}} \exp \left(\sum_u \frac{1}{2} \sum_{a>l} \left(\sqrt{\frac{\mathrm{i}\hat{\mu}_u}{N}} \sum_i x_i^u \xi_i^a \right)^2 \right)\right)\Bigg).\end{aligned} \tag{S5.5}$$

To compute the last term, we can integrate over disorder by factorising over patterns a and introducing a Gaussian

integral ($\int Dz \exp(az) = \exp(a^2/2)$) to obtain

$$\begin{aligned}
\frac{1}{2^{N(M-l)}}\sum_{\boldsymbol{\xi}^{a>l}}\exp\left(\sum_{a>l}\sum_{u}\left(\frac{1}{2}\sqrt{\frac{\mathrm{i}\hat{\mu}_u}{N}}\sum_i x_i^u\xi_i^a\right)^2\right) &= \frac{1}{2^{N(M-l)}}\prod_{a>l}\sum_{\boldsymbol{\xi}^a}\exp\left(\sum_u\frac{1}{2}\left(\sqrt{\frac{\mathrm{i}\hat{\mu}_u}{N}}\sum_i x_i^u\xi_i^a\right)^2\right)\\
&= \prod_{a>l}\frac{1}{2^{N(M-l)}}\sum_{\boldsymbol{\xi}^a}\int D\boldsymbol{z}\exp\left(\sum_u z_u\sqrt{\frac{\mathrm{i}\hat{\mu}_u}{N}}\sum_i x_i^u\xi_i^a\right)\\
&= \frac{1}{2^{N(M-l)}}\left(\int D\boldsymbol{z}\exp\left(\sum_i \ln 2\cosh\left(\sum_u\sqrt{\frac{\mathrm{i}\hat{\mu}_u}{N}}z_u x_i^u\right)\right)\right)^{M-l}\\
&= \left(\int D\boldsymbol{z}\exp\left(\sum_i \ln\cosh\left(\sum_u\sqrt{\frac{\mathrm{i}\hat{\mu}_u}{N}}z_u x_i^u\right)\right)\right)^{M-l}\\
&= \left(\int D\boldsymbol{z}\exp\left(\frac{1}{2}\sum_{u,v}z_u z_v\sqrt{\mathrm{i}\hat{\mu}_u\mathrm{i}\hat{\mu}_v}\frac{1}{N}\sum_i x_i^u x_i^v\right)\right)^{M-l}, \qquad \text{(S5.6)}
\end{aligned}$$

where the $\ln\cosh$ term was approximated assuming a large N ($\ln\cosh x \approx \ln(1+x^2/2) \approx x^2/2$ for $x \ll 1$). By introducing an additional delta integral for order parameters q_{uv} (assuming $q_{uu} = 1$) and applying $\exp\ln$, one can re-express the last term (assuming $M - l \approx N\alpha$ near saturation) as

$$\begin{aligned}
&\exp\left(N\alpha\ln\int D\boldsymbol{z}\exp\left(\frac{1}{2}\sum_{u,v}z_u z_v\sqrt{\mathrm{i}\hat{\mu}_u\mathrm{i}\hat{\mu}_v}\frac{1}{N}\sum_i x_i^u x_i^v\right)\right)\\
&= \frac{1}{(2\pi)^{\frac{n(n-1)}{2}}}\int d\boldsymbol{q}d\hat{\boldsymbol{q}}\exp\left(N\alpha\ln\int D\boldsymbol{z}\exp\left(\frac{1}{2}\sum_{u,v}z_u z_v\sqrt{\mathrm{i}\hat{\mu}_u\mathrm{i}\hat{\mu}_v}q_{uv}\right) - \sum_{u<v}\mathrm{i}\hat{q}_{uv}\left(q_{uv} - \frac{1}{N}\sum_i x_i^u x_i^v\right)\right)\\
&= \frac{1}{(2\pi)^{\frac{n(n-1)}{2}}}\int d\boldsymbol{q}d\hat{\boldsymbol{q}}\exp\left(-\frac{1}{2}N\alpha\ln|\Lambda| - \sum_{u<v}\mathrm{i}\hat{q}_{uv}\left(q_{uv} - \frac{1}{N}\sum_i x_i^u x_i^v\right)\right), \qquad \text{(S5.7)}
\end{aligned}$$

where $\Lambda_{uv} = \delta_{uv}(1 - \sqrt{\mathrm{i}\hat{\mu}_u\mathrm{i}\hat{\mu}_v}q_{uv}) + (1-\delta_{uv})\left(-\sqrt{\mathrm{i}\hat{\mu}_u\mathrm{i}\hat{\mu}_v}q_{uv}\right) = \delta_{uv} - \sqrt{\mathrm{i}\hat{\mu}_u\mathrm{i}\hat{\mu}_v}q_{uv}$. Then, the configurational average is found to be

$$\begin{aligned}
\langle\!\langle Z^n\rangle\!\rangle &= \frac{1}{(2\pi)^{(l+1)n+\frac{n(n-1)}{2}}}\int d\boldsymbol{\pi}\sum_{\boldsymbol{x}}\exp\left(\frac{1}{\gamma}\sum_u\ln\left(1+\gamma\beta\left(\sum_{b\le l}\left(NH_b m_b^u + \frac{JN}{2}(m_b^u)^2\right) + J\mu_u - N\frac{J\alpha}{2}\right)\right)\right.\\
&\quad\left. - \sum_{u,b\le l}\mathrm{i}\hat{m}_b^u\left(m_b^u - \frac{1}{N}\sum_i x_i^u\xi_i^b\right) - \sum_u\mathrm{i}\hat{\mu}_u\mu_u - \frac{1}{2}N\alpha\ln|\boldsymbol{\Lambda}| - \sum_{u<v}\mathrm{i}\hat{q}_{uv}\left(q_{uv} - \frac{1}{N}\sum_i x_i^u x_i^v\right)\right)\\
&= \frac{1}{(2\pi)^{(l+1)n+\frac{n(n-1)}{2}}}\int d\boldsymbol{\pi}\exp\left(\frac{1}{\gamma}\sum_u\ln\left(1+\gamma\beta\left(\sum_{b\le l}\left(NH_b m_b^u + \frac{JN}{2}(m_b^u)^2\right) + J\mu_u - N\frac{J\alpha}{2}\right)\right)\right.\\
&\quad\left. - \sum_{u,b\le l}\mathrm{i}\hat{m}_b^u m_b^u - \sum_u\mathrm{i}\hat{\mu}_u\mu_u - \frac{1}{2}N\alpha\ln|\boldsymbol{\Lambda}| - \sum_{u<v}\mathrm{i}\hat{q}_{uv}q_{uv} + \ln\sum_{\boldsymbol{x}}\exp L\right), \qquad \text{(S5.8)}
\end{aligned}$$

where $d\boldsymbol{\pi} \coloneqq d\boldsymbol{m}d\hat{\boldsymbol{m}}d\boldsymbol{\mu}d\hat{\boldsymbol{\mu}}d\boldsymbol{q}d\hat{\boldsymbol{q}}$ has been adopted for readability, with

$$L = \sum_{u,b\le l}\mathrm{i}\hat{m}_b^u\frac{1}{N}\sum_i x_i^u\xi_i^b + \sum_{u<v}\mathrm{i}\hat{q}_{uv}\frac{1}{N}\sum_i x_i^u x_i^v \qquad \text{(S5.9)}$$

carrying all remaining x_i dependent terms to be summed. The saddle-node solution is given by

$$\begin{aligned}
\mathrm{i}\hat{m}_a^u &= N\beta_u'(H_a + Jm_a^u),\\
\mathrm{i}\hat{\mu}_u &= \beta_u' J,\\
m_a^u &= \frac{1}{N}\sum_i \xi_i^a \left\langle x_i^u \right\rangle_L := \frac{1}{N}\sum_i \xi_i^a \frac{\sum_{\boldsymbol{x}} x_i^u \exp L}{\sum_{\boldsymbol{x}} \exp L},\\
q_{uv} &= \frac{1}{N}\sum_i \left\langle x_i^u x_i^v \right\rangle_L = \frac{1}{N}\sum_i \frac{\sum_{\boldsymbol{x}} x_i^u x_i^v \exp L}{\sum_{\boldsymbol{x}} \exp L},\\
\mathrm{i}\hat{q}_{uv} &= N\alpha\sqrt{\mathrm{i}\hat{\mu}_u \mathrm{i}\hat{\mu}_v}\left\langle z_u z_v \right\rangle_* = N\alpha J^2 \beta_u' \beta_v' r_{uv},\\
\mu_u &= \frac{1}{2}N\alpha \sum_v \sqrt{\frac{\beta_v'}{\beta_u'}} q_{uv}\left\langle z_u z_v \right\rangle_* = \frac{1}{2}N\alpha J\sum_v \sqrt{\beta_u'\beta_v'} q_{uv} r_{uv},\\
\beta_u' &= \frac{\beta}{1+\gamma N\beta \frac{1}{2}\left(\sum_{b\le l}(2H_b + Jm_b^u)m_b^u + \alpha J^2 \sum_v \sqrt{\beta_u'\beta_v'} q_{uv} r_{uv} - J\alpha\right)},\\
r_{uv} &= \frac{1}{J\sqrt{\beta_u'\beta_v'}}\left\langle z_u z_v \right\rangle_* := \frac{1}{J\sqrt{\beta_u'\beta_v'}} \frac{\int D\boldsymbol{z} z_u z_v \exp\left(\frac{1}{2}\sum_{u,v} z_u z_v \left(\sqrt{\beta_u'\beta_v'} J q_{uv}\right)\right)}{\int D\boldsymbol{z} \exp\left(\frac{1}{2}\sum_{u,v} z_u z_v \left(\sqrt{\beta_u'\beta_v'} J q_{uv}\right)\right)},
\end{aligned} \tag{S5.10}$$

where the operator $\left\langle f(\boldsymbol{x})\right\rangle_*$ defined above coincides with regular averages once integration is performed. This results in

$$\begin{aligned}
\ln\langle\!\langle Z^n \rangle\!\rangle = &\frac{1}{\gamma}\sum_u \ln\left(1+\gamma\beta\left(\sum_{b\le l}\left(NH_b m_b^u + \frac{JN}{2}\left(m_b^u\right)^2\right) + J\mu_u - N\frac{J\alpha}{2}\right)\right)\\
&-\sum_{u,b\le l} N\beta_u'(H_b + Jm_b^u)m_b^u - \sum_u \beta_u' J\mu_u - \frac{1}{2}N\alpha\ln|\boldsymbol{\Lambda}| - \sum_{u<v} N\alpha\beta_u'\beta_v' J^2 r_{uv} q_{uv}\\
&+\ln\sum_{\boldsymbol{x}} \exp L
\end{aligned} \tag{S5.11}$$

with L being given (due to (S5.9)) by

$$L = \sum_{u,b\le l}\beta_u'(H_b + Jm_b^u)\sum_i x_i^u \xi_i^b + J^2\alpha\sum_{u<v}\beta_u'\beta_v' r_{uv}\sum_i x_i^u x_i^v. \tag{S5.12}$$

2. Replica symmetry

The replica symmetry ansatz allows us to simplify order parameters m_b^u, q_{uv}, r_{uv} (for $u \neq v$), and r_{uu} to homogeneous values m_b, q, r, and R (note that $q_{uu} = 1$). Assuming a normalised curvature parameter $\gamma = \frac{\gamma'}{N\beta}$, we obtain

$$\begin{aligned}
\frac{1}{n}\ln\langle\!\langle Z^n \rangle\!\rangle = &\frac{N\beta}{\gamma'}\ln\left(1+\gamma'\left(\sum_{b\le l}\left(H_b m_b + \frac{J}{2}\left(m_b\right)^2\right) + \frac{J}{N}\mu - \frac{J\alpha}{2}\right)\right)\\
&-\sum_{b\le l} N\beta_u'(H_b + Jm_b)m_b - \beta' J\mu - \frac{1}{2n}N\alpha\ln|\boldsymbol{\Lambda}|\\
&-\frac{1}{2}(n-1)N\alpha\beta'^2 J^2 rq + \frac{1}{n}\ln\sum_{\boldsymbol{x}}\exp L,
\end{aligned} \tag{S5.13}$$

where $J\beta'\mu = \frac{1}{2}N\alpha(J\beta')^2(R + (n-1)qr)$ and

$$L = \beta'\sum_{u,b\le l}(H_b + Jm_b)\sum_i x_i^u \xi_i^b + \sum_{u<v}\beta'^2 J^2\alpha r\sum_i x_i^u x_i^v. \tag{S5.14}$$

We obtain

$$\begin{aligned}
\ln\sum_{\boldsymbol{x}}\exp L &= \ln\sum_{\boldsymbol{x}}\exp\left(\sum_{u,b\le l}\beta'(H_b+Jm_b)\sum_i \xi_i^b x_i^u + (\beta' J)^2\,\alpha r\sum_i\sum_{u<v}x_i^u x_i^v\right)\\
&= \ln\prod_i\sum_{\boldsymbol{x}_i}\exp\left(\beta'\sum_{u,b\le l}(H_b+Jm_b)\xi_i^b x_i^u + \frac{1}{2}\left(\beta' J\sqrt{\alpha r}\sum_u x_i^u\right)^2 - \frac{1}{2}n(\beta' J)^2\alpha r\right)\\
&= \ln\prod_i\int Dz\sum_{\boldsymbol{x}_i}\exp\left(\beta'\sum_{u,b\le l}(H_b+Jm_b)\xi_i^b x_i^u + \beta' J\sqrt{\alpha r}z\sum_u x_i^u - \frac{1}{2}n(\beta' J)^2\alpha r\right)\\
&= \sum_i\ln\int Dz\exp\left(n\ln\left(2\cosh\left(\beta'\sum_{b\le l}(H_b+Jm_b)\xi_i^b + \beta' J\sqrt{\alpha r}z\right)\right) - \frac{1}{2}n(\beta' J)^2\alpha r\right)\\
&= \sum_i n\int Dz\ln\left(2\cosh\left(\beta'\sum_{b\le l}(H_b+Jm_b)\xi_i^b + \beta' J\sqrt{\alpha r}z\right)\right) - \frac{1}{2}nN\,(\beta' J)^2\,\alpha r,
\end{aligned} \tag{S5.15}$$

where in the last step we assum a small value of n (as we will apply later the limit $n\to 0$).

Overlaps corresponding to non-nominated patterns To apply the replica symmetry argument near the $n\to 0$ limit, we know from (S5.10) that the covariance matrix of $\beta' J r_{uv}$ corresponds to the inverse of $\boldsymbol{\Lambda}$ (with $\Lambda_{uv} = \delta_{uv} - \beta' J(\delta_{uv} + (1-\delta_{uv})q) = \delta_{uv}(1-\beta' J(1-q)) - \beta' Jq$). Under the replica symmetry assumption, the inverse is given via the Sherman-Morrison formula,

$$\Lambda_{uv}^{-1} = \frac{1}{1-\beta' J(1-q)}\left(\delta_{uv} + \frac{\beta' Jq}{1-\beta' J(1-q) - n\beta' Jq}\right), \tag{S5.16}$$

evaluating the limit $n=0$,

$$\beta' J r_{uv} = \delta_{uv}\frac{1-\beta' J(1-2q)}{(1-\beta' J(1-q))^2} + (1-\delta_{uv})\frac{\beta' Jq}{(1-\beta' J(1-q))^2}. \tag{S5.17}$$

Identifying R and r as the respective diagonal and off diagonal parts of r_{uv}, we can determine

$$\mu \xrightarrow{n\to 0} \frac{1}{2}N\alpha\beta' J(R-qr)\,. \tag{S5.18}$$

This leads to an effective inverse temperature,

$$\beta' = \frac{\beta}{1+\gamma'\frac{1}{2}\left(\sum_{b\le l}(2H_b+Jm_b)m_b + \alpha J\left(\frac{1-\beta' J(1-q)^2}{(1-\beta' J(1-q))^2} - 1\right)\right)}. \tag{S5.19}$$

Replica symmetric solution We have

$$\begin{aligned}
\frac{1}{n}\ln\langle\!\langle Z^n\rangle\!\rangle = \frac{N\beta}{\gamma'}&\ln\left(1+\gamma'\left(\sum_{b\le l}\left(H_b m_b + \frac{J}{2}(m_b)^2\right) + \frac{J}{N}\mu - \frac{J\alpha}{2}\right)\right)\\
&- N\beta'\sum_{b\le l}(H_b+Jm_b)m_b - \beta' J\mu - \frac{1}{2n}N\alpha\ln|\Lambda| - \frac{1}{2}N\alpha(n-1)(J\beta')^2 rq\\
&+\sum_i\int Dz\ln\left(2\cosh\left(\beta'\sum_{b\le l}(H_b+Jm_b)\xi_i^b + \beta' J\sqrt{\alpha r}z\right)\right) - \frac{1}{2}N\,(\beta' J)^2\,\alpha r.
\end{aligned} \tag{S5.20}$$

Near the limit of $n\to 0$, we can approximate

$$\begin{aligned}
\ln|\Lambda| &= \ln(1-\beta' J(1-q) - \beta' Jqn) + (n-1)\ln(1-\beta' J(1-q))\\
&= n\left(\ln(1-\beta' J(1-q)) - \frac{\beta' Jq}{1-\beta' J(1-q)}\right).
\end{aligned} \tag{S5.21}$$

Using the approximation in (S5.21), extremisation of (S5.20) with respect to H_a yields:

$$m_a = \frac{1}{N}\sum_i \xi_i^a \int Dz \tanh\left(\beta' \sum_{b\leq l}(H_b + Jm_b)\xi_i^b + \beta' J\sqrt{\alpha r}z\right). \tag{S5.22}$$

Similarly, extremisation with respect to r yields

$$\frac{\beta'^2 J^2}{2} N\alpha(1-q) = N\beta' J\frac{1}{2}\sqrt{\frac{\alpha}{r}}\int Dz\, z\tanh\left(\beta' \sum_{b\leq l}(H_b + Jm_b)\xi_i^b + \beta' J\sqrt{\alpha r}z\right), \tag{S5.23}$$

which, by applying the partial integration, results in

$$q = \int Dz \tanh^2\left(\beta'\left(\sum_{b\leq l}(H_b + Jm_b)\xi_i^b + J\sqrt{\alpha r}z\right)\right). \tag{S5.24}$$

We can observe that for $\alpha = 0$, we recover previous results in (S3.10). In addition, we obtain Eqs. 21 and 22 for one memory pattern ($l = 1$) given by all positive unity values and $H_b = 0$. Further, for $\gamma = 0$, $\beta' = \beta$, and the solution corresponds to the Hopfield model near saturation [58, 62].

Notice that in the limit $J \to 0$ and $\boldsymbol{H} = \boldsymbol{0}$, we obtain

$$\beta' J^2\mu = \frac{1}{2}NJ\alpha(1 + \beta' J(1-q^2)) - N\frac{J\alpha}{2}, \tag{S5.25}$$

$$r = q, \tag{S5.26}$$

$$\beta' = \frac{\beta}{1 + \gamma N\beta\frac{1}{2}\alpha\beta' J^2(1-q^2)}. \tag{S5.27}$$

For a scaled value of spin coupling strength, defined as $\tilde{J}$ such that $\alpha = 1$ and $\gamma' = \gamma N\beta\frac{J^2}{\tilde{J}^2}$, the equations above recover the solution for the curved Sherrington-Kirkpatrick model in Eqs. (S6.17-S6.19).

3. AT-instability line

This section probes how the deformation of the statistics modifies the boundary below which we may no longer rely on replica symmetry. Following [58] let us then consider small fluctuations η_{uv} around the replica symmetric expressions for q_{uv} and its conjugated pair.

$$q_{uv} \mapsto q_{uv}^{\mathrm{RS}} + \eta_{uv} := \delta_{uv} + q(1-\delta_{uv}) + \eta_{uv} \tag{S5.28}$$

with $\eta_{uv} = \eta_{vu}$, vanishing diagonal elements, and $\sum_u \eta_{uv} = 0$. We are ultimately interested in the free energy difference,

$$\tfrac{1}{N}\Delta\varphi_\gamma := \tfrac{1}{N}[\varphi_\gamma(m^{\mathrm{RS}}, q_{uv}, \hat{q}_{uv}) - \varphi_\gamma(m^{\mathrm{RS}}, q_{uv}^{\mathrm{RS}}, \hat{q}_{uv}^{\mathrm{RS}})]. \tag{S5.29}$$

One should be mindful that β'_u may be affected by fluctuations. The effective inverse temperature β'_u depends on μ, which is itself a function of both q_{uv} and $\hat{q}_{uv}$. One can anticipate that $\hat{q}_{uv}$, and thereby β'_u, will be a polynomial in η_{uv}. The coefficients of the perturbative expansion of β'_u are determined by replica-symmetric parameters, and hence its index structure follows from the properties of η_{uv} rule out linear contributions. Without loss of generality, we have up to the second order,

$$\beta' = \beta'_0 + \beta'_1\sum_v \eta_{uv}^2 + \mathcal{O}(\boldsymbol{\eta}^3), \tag{S5.30}$$

for some β'_1 to be determined and β'_0 being its RS-value, which only distinguishes between diagonal and off-diagonal components. Let us first recall that β'_0 at (S5.10) under the RS assumption becomes,

$$\beta'_0 = \frac{\beta}{1 + \frac{1}{2}\gamma'(\bar{m} + \alpha\beta'_0 J^2(R - qr) - J\alpha)} \tag{S5.31}$$

with $\bar{m} = 2\sum_{b\leq l}\left(H_b m_b + \frac{J}{2}(m_b)^2\right)$ adopted for brevity. Solving for β_0' from the expression above leads to,

$$\beta_0' = \frac{2 - J\alpha\gamma' + \gamma'\bar{m} \mp \sqrt{(2 - J\alpha\gamma' + \gamma'\bar{m})^2 + 8\beta J^2\alpha\gamma'(R - qr)}}{2\alpha\gamma'(qr - R)}. \tag{S5.32}$$

To resolve how its conjugate, $\hat{q}_{uv}$, transforms, we inspect the two-point functions $\langle z_u z_v\rangle_*$ upon small perturbations of the order parameter η_{uv},

$$\langle z_u z_v\rangle_* \mapsto \frac{\langle z_a z_b\rangle_* + \frac{1}{2}\sum_{c,d}\langle z_a z_b z_c z_d\rangle_* \Lambda_{cd}}{1 + \frac{1}{2}\sum_{c,d}\langle z_c z_d\rangle_* \Lambda_{cd}} \simeq \langle z_a z_b\rangle_* + \frac{1}{2}\sum_{c,d}\Lambda_{cd}[\langle z_a z_b z_c z_d\rangle_* - \langle z_a z_b\rangle_* \langle z_c z_d\rangle_*] \tag{S5.33}$$

with

$$\Lambda_{cd} = \beta_0' J\eta_{cd} + \beta_1' J\sum_s(\eta_{cs}^2 + \eta_{ds}^2)[\delta_{cd} + q(1 - \delta_{cd})]. \tag{S5.34}$$

This implies that $\hat{\eta}_{cd}$, defined as the change of the two point function, and thereby $\hat{q}_{uv}$ —conjugate to q_{uv}— carries a dependence of second order in fluctuations parametrised by β_1'.

$$\begin{aligned}\hat{q}_{uv} &= -\mathrm{i}N\alpha\sqrt{\beta_u'\beta_v'}J\langle z_u z_v\rangle_* \\ &\to -\mathrm{i}N\alpha J\left(\beta_0' + \frac{1}{2}\beta_1'\sum_s[\eta_{us}^2 + \eta_{vs}^2]\right)[\langle z_u z_v\rangle_* + \hat{\eta}_{uv}] \\ &= \hat{q}_{uv}^{\mathrm{RS}} - \mathrm{i}N\alpha J\left(\beta_0'\hat{\eta}_{uv} + \frac{1}{2}\beta_1'\sum_s\langle z_u z_v\rangle_*[\eta_{us}^2 + \eta_{vs}^2]\right).\end{aligned} \tag{S5.35}$$

Here β_1' can be obtained from the expression for β_u' at (S5.10) exploring $\sum_v \beta_v' q_{uv} r_{uv}$ under perturbations.

$$\begin{aligned}\sum_v q_{uv}\langle z_u z_v\rangle_* &\mapsto \sum_v(q_{uv}^{\mathrm{RS}} + \eta_{uv})(\langle z_u z_v\rangle_* + \hat{\eta}_{uv}) \\ &= \beta_0' J(R - rq) + \sum_v(q_{uv}^{\mathrm{RS}}\hat{\eta}_{uv} + \langle z_u z_v\rangle_*\eta_{uv} + \eta_{uv}\hat{\eta}_{uv}) \\ &= \beta_0' J(R - rq) + \frac{1}{2}\beta_1'\sum_{v,s,c,d}q_{uv}^{\mathrm{RS}}q_{cd}^{\mathrm{RS}}g_{uvcd}(\eta_{cs}^2 + \eta_{ds}^2) + \frac{1}{2}\beta_0'\sum_{v,c,d}g_{uvcd}\eta_{uv}\eta_{cd},\end{aligned} \tag{S5.36}$$

where the first term results from its RS-valued part and

$$g_{abcd} = \langle z_a z_b z_c z_d\rangle_* - \langle z_a z_b\rangle_* \langle z_c z_d\rangle_* = \langle z_a z_c\rangle_* \langle z_b z_d\rangle_* + \langle z_a z_d\rangle_* \langle z_b z_c\rangle_* \tag{S5.37}$$

has been adopted for brevity. The four-point function can be reduced via Wick Theorem to products of two-point functions. Linear terms in fluctuations coupled to RS-terms vanish with the sum as expected. It should be noted that unlike the flat case, the $\sum_s \hat{\eta}_{us} = 0$ property no longer holds due to quadratic terms in perturbations.

Let us now evaluate the sums at (S5.36),

$$\begin{aligned}\sum_{v,s,c,d}q_{uv}^{\mathrm{RS}}q_{cd}^{\mathrm{RS}}g_{uvcd}(\eta_{cs}^2 + \eta_{ds}^2) &= \sum_{v,s,c,d}q_{uv}^{\mathrm{RS}}q_{cd}^{\mathrm{RS}}(\langle z_u z_c\rangle_* \langle z_v z_d\rangle_* + \langle z_u z_d\rangle_* \langle z_v z_c\rangle_*)(\eta_{cs}^2 + \eta_{ds}^2) \\ &= (\beta_0' J)^2\sum_{v,s,c,d}q_{uv}^{\mathrm{RS}}q_{cd}^{\mathrm{RS}}(r_{uc}^{\mathrm{RS}}r_{vd}^{\mathrm{RS}} + r_{ud}^{\mathrm{RS}}r_{vc}^{\mathrm{RS}})(\eta_{cs}^2 + \eta_{ds}^2)\end{aligned} \tag{S5.38}$$

from here, we break the sums into diagonal and off-diagonal contributions

$$\begin{aligned}&= 4(\beta_0' J)^2\sum_{v,s,c}q_{uv}^{\mathrm{RS}}r_{uc}^{\mathrm{RS}}r_{vc}^{\mathrm{RS}}\eta_{cs}^2 + 2q(\beta_0' J)^2\sum_{v,s,c\neq d}q_{uv}^{\mathrm{RS}}(r_{uc}^{\mathrm{RS}}r_{vd}^{\mathrm{RS}} + r_{ud}^{\mathrm{RS}}r_{vc}^{\mathrm{RS}})\eta_{cs}^2 \\ &= 4(\beta_0' J)^2\sum_{s,c}r_{uc}^{\mathrm{RS}2}\eta_{cs}^2 + 4q(\beta_0' J)^2\sum_{v\neq u,s,c}r_{uc}^{\mathrm{RS}}r_{vc}^{\mathrm{RS}}\eta_{cs}^2 + 4q(\beta_0' J)^2\sum_{s,c\neq d}r_{ud}^{\mathrm{RS}}r_{uc}^{\mathrm{RS}}\eta_{cs}^2 \\ &\quad + 2q^2(\beta_0' J)^2\sum_{s,v\neq u,c\neq d}(r_{uc}^{\mathrm{RS}}r_{vd}^{\mathrm{RS}} + r_{ud}^{\mathrm{RS}}r_{vc}^{\mathrm{RS}})\eta_{cs}^2.\end{aligned} \tag{S5.39}$$

Evaluation of $r_{ud}^{\mathrm{RS}} = R\delta_{ud} + r(1-\delta_{ud})$ yields a polynomial we will call for the moment $f(q, \beta_0', r, R)$, and so the expression may be succinctly written as $f(q, \beta_0', r, R) \sum_s \eta_{us}^2$. More importantly,

$$\begin{aligned}\sum_{v,c,d} g_{uvcd}\eta_{uv}\eta_{cd} &= (\beta_0' J)^2 \sum_{v,c,d} (r_{uc}^{\mathrm{RS}} r_{vd}^{\mathrm{RS}} + r_{ud}^{\mathrm{RS}} r_{vc}^{\mathrm{RS}})\eta_{uv}\eta_{cd} \\ &= (\beta_0' J)^2 \sum_{v\neq u, c\neq d} (r_{uc}^{\mathrm{RS}} r_{vd}^{\mathrm{RS}} + r_{ud}^{\mathrm{RS}} r_{vc}^{\mathrm{RS}})\eta_{uv}\eta_{cd} \\ &= 2(\beta_0' J)^2 \sum_{v\neq u, c\neq d} r_{uc}^{\mathrm{RS}} r_{vd}^{\mathrm{RS}} \eta_{uv}\eta_{cd} = 2(\beta_0' J)^2 \sum_{c\neq d} r_{uc}^{\mathrm{RS}} \left(\sum_{v\neq u} r_{vd}^{\mathrm{RS}} \eta_{uv} \right) \eta_{cd} = 0. \end{aligned} \tag{S5.40}$$

Now we can solve for β_1', expanding the self-consistent equation,

$$\begin{aligned}\beta_0' + \beta_1' \sum_s \eta_{vs}^2 &\simeq \beta_0' \left(1 - \frac{1}{4\Gamma}\gamma'\alpha J \left(\beta_1' \sum_{v,s,c,d} q_{uv}^{\mathrm{RS}} q_{cd}^{\mathrm{RS}} g_{uvcd}(\eta_{cs}^2 + \eta_{ds}^2) + \beta_0' \sum_{v,c,d} g_{uvcd}\eta_{uv}\eta_{cd} \right) \right) \\ \beta_1' \sum_s \eta_{vs}^2 &\simeq -\frac{1}{4\Gamma}\gamma'\alpha J \beta_1' f(q, \beta_0', r, R) \sum_s \eta_{us}^2, \end{aligned} \tag{S5.41}$$

where Γ is defined as denominator of the expression for β' at (24),

$$\Gamma := 1 + \frac{1}{2}\gamma' \left(Jm^2 + \alpha J(\beta'(R - qr) - 1) \right) \tag{S5.42}$$

seeming to imply that β' does not seem to be altered at the second order of perturbations, and perhaps the effects are only seen at higher orders. This greatly simplifies the analysis onwards; $\hat{q}_{uv}$ and $\hat{\eta}_{uv}$ are now first order in η_{uv}, the latter reduced to $\hat{\eta}_{uv} = \beta_0'^2 (R-r)^2 \eta_{uv}$. The same expression for $\gamma = 0$ up to a scaled inverse temperature is recovered. Let us focus on the free energy difference,

$$\begin{aligned}\frac{1}{N}\Delta\varphi_\gamma \ni \frac{1}{2N}\sum_{u,v}(\mathrm{i}\hat{q}_{uv} q_{uv} - \mathrm{i}\hat{q}_{uv}^{\mathrm{RS}} q_{uv}^{\mathrm{RS}}) &= \frac{\mathrm{i}}{2N}\operatorname{Tr}[\hat{q}_{uv}^{\mathrm{RS}}\eta_{uv}] + \frac{1}{2}\alpha(\beta' J)^2 \operatorname{Tr}[\hat{\eta}_{uv}\hat{q}_{uv}^{\mathrm{RS}} + \hat{\eta}_{uv}\eta_{uv}] \\ &= \frac{1}{2}\alpha(\beta' J)^2 \operatorname{Tr}[\hat{\eta}_{uv}\eta_{uv}]. \end{aligned} \tag{S5.43}$$

The trace is understood over replica indices. Notice that despite the seemly different overall coefficient and sign [58], this is just an artifact of the convention on the introduction of the deltas; these expressions are equivalent. The diagonal terms are included to make up the trace vanish as constants at the free energy difference.

The determinants transform as,

$$\frac{1}{N}\Delta\varphi_\gamma \ni \ln \frac{\left|1 - \beta'(\boldsymbol{q}^{\mathrm{RS}} + \boldsymbol{\eta})\right|}{\left|1 - \beta' \boldsymbol{q}^{\mathrm{RS}}\right|} = -\frac{1}{2}\frac{\beta_0'^2}{[1 - \beta_0'(1-q)]^2}\operatorname{Tr}\boldsymbol{\eta}^2 + \mathcal{O}(\boldsymbol{\eta}^3). \tag{S5.44}$$

There is a contribution from the $L(x_i)$-function and the logarithm that results from the deformation. First the L-term contribution

$$\frac{1}{N}\Delta\varphi_\gamma \ni \ln \frac{\sum_{\boldsymbol{x}} \exp L(m^{\mathrm{RS}}, q_{uv}, \hat{q}_{uv}, x_i)}{\sum_{\boldsymbol{x}} \exp L(m^{\mathrm{RS}}, q_{uv}^{\mathrm{RS}}, \hat{q}_{uv}^{\mathrm{RS}}, x_i)}, \tag{S5.45}$$

which basically amounts to, after expansion,

$$\begin{aligned}\ln \sum_{\boldsymbol{x}} \exp L(m^{\mathrm{RS}}, q_{uv}, \hat{q}_{uv}, x_i) \simeq \ln \prod_i \sum_{\boldsymbol{x}_i} \exp \Lambda^{\mathrm{RS}}(x_i) \Big[1 + \alpha J^2 \sum_{u,v} x_i^u \hat{\eta}_{uv} x_i^v \sum_{u,v} \beta_{0u}' \eta_{uv} \beta_{1v}' \\ \frac{1}{2}\alpha J^2 \beta_0'^2 \sum_{u,v} x_i^u \hat{\eta}_{uv} x_i^v + \frac{1}{8}\alpha^2 (\beta_0' J)^4 \sum_{u,v} (x_i^u \hat{\eta}_{uv} x_i^v)^2 \Big]. \end{aligned} \tag{S5.46}$$

However, as we concluded previously, β_u' does not have a second-order term.

$$= \ln \prod_i \sum_{\boldsymbol{x}_i} \exp \Lambda^{\mathrm{RS}}(x_i) \left[1 + \frac{1}{2}\alpha(\beta_0' J)^2 \sum_{u,v} x_i^u \hat{\eta}_{uv} x_i^v + \frac{1}{8}\alpha^2 (\beta_0' J)^4 \sum_{u,v} (x_i^u \hat{\eta}_{uv} x_i^v)^2 \right], \tag{S5.47}$$

where $\Lambda^{\mathrm{RS}}(x_i)$ has been defined as the argument of the exponential at (S5.15). Once again, the trace can be recovered at the fluctuation terms can be recovered noticing that $\eta_{uu} = 0$. The denominator eventually cancels off the contributions from $\Lambda^{\mathrm{RS}}(x_i)$, and we are left with the part from the squared brackets. The first term can be recognised as the average defined at the saddle-node solution for q_{ab} (S5.10). Finally, the logarithm that results from the deformation of the statistics and μ,

$$\frac{1}{N}\Delta\varphi_\gamma \ni \frac{\beta}{\gamma'}\ln\left(\frac{\beta'_{\mathrm{RS}}}{\beta'}\right) - \beta' J\Delta\mu = 0 \tag{S5.48}$$

are up to second order in perturbations, invariant, and hence do not contribute to the free energy difference. Following the derivation of $\Delta\varphi_\gamma$ for $\gamma \to 0$ at [58], we may determine,

$$\begin{aligned}\frac{1}{N}\Delta\varphi_\gamma = \frac{1}{\beta'_0 n}\Bigg(& -\frac{1}{4}\frac{\alpha\beta_0'^2}{[1-\beta'_0(1-q)]_+^2}\operatorname{Tr}\boldsymbol{\eta}^2 + \frac{1}{2}\alpha\beta_0'^4(R-r)^2\operatorname{Tr}\boldsymbol{\eta}^2 \\ & -\frac{1}{8}\alpha^2\beta_0'^8(R-r)^4\sum_{a,b,c,d}\eta_{ab}\eta_{cd}G_{abcd}\Bigg),\end{aligned} \tag{S5.49}$$

where

$$\begin{aligned}G_{abcd} = {} & \delta_{ac}\delta_{bd} + \delta_{ad}\delta_{bc} + G_4(1-\delta_{ac})(1-\delta_{bd})(1-\delta_{ad})(1-\delta_{bc}) \\ & + G_2(\delta_{ac}(1-\delta_{bd}) + \delta_{bd}(1-\delta_{ac}) + \delta_{ad}(1-\delta_{bc}) + \delta_{bc}(1-\delta_{ad})),\end{aligned} \tag{S5.50}$$

with

$$G_\ell = \frac{1}{N}\sum_i \frac{\int Dz\tanh^\ell \beta'_0[\sum_{b\le l}(H_b + Jm_b)\xi_i^b + z\sqrt{\alpha r}]\cosh^n \beta'_0[\sum_{b\le l}(H_b + Jm_b)\xi_i^b + z\sqrt{\alpha r}]}{\int Dz\cosh^n \beta'_0[\sum_{b\le l}(H_b + Jm_b)\xi_i^b + z\sqrt{\alpha r}]}. \tag{S5.51}$$

leading to a condition

$$(1+\beta'_0(1-q))^2 > \alpha\beta_0'^2\frac{1}{N}\sum_i \xi_i^a\int Dz\cosh^{-4}\beta'_0\left(\sum_{b\le l}(H_b + Jm_b)\xi_i^b + J\sqrt{\alpha r}z\right) \tag{S5.52}$$

equivalent to that of the flat model (see [58], equation (121)) with a rescaled inverse temperature.

Supplementary Note 6: Curved Sherrington-Kirkpatrick model

We start with a simple case in which the system is encoding one pattern on a background of zero-average Gaussian weights. This can be represented by $J_{ij} = J_0/N\xi_i\xi_j + J/\sqrt{N}z_{ij}$, with z_{ij} random coupling values distributed as $\mathcal{N}(0,1)$. Assuming the content of the $[]_+$ operator is positive, we want to compute the configurational average

$$\langle\!\langle \varphi_\gamma \rangle\!\rangle = \int D\boldsymbol{z} \ln \sum_{\boldsymbol{x}} \left(1 + \gamma\beta\left(\frac{J_0}{N}\sum_{i<j} x_i\xi_i\xi_j x_j + \frac{J}{\sqrt{N}}\sum_{i<j} z_{ij}x_i x_j\right)\right)^{1/\gamma} \tag{S6.1}$$
$$= \int D\boldsymbol{z} \ln \sum_{\boldsymbol{x}} \left(1 + \gamma\beta\left(\frac{NJ_0}{2}\left(\frac{1}{N}\sum_i x_i\xi_i\right)^2 - \frac{J_0}{2} + \frac{J}{\sqrt{N}}\sum_{i<j} z_{ij}x_i x_j\right)\right)^{1/\gamma},$$

where we define $D\boldsymbol{z} = \prod_{i<j} dz_{ij}\frac{1}{\sqrt{2\pi}}\exp\left(-\frac{1}{2}z_{ij}^2\right)$.

Defining $\varphi_\gamma = \ln Z$, we can apply the replica method

$$\langle\!\langle \varphi_\gamma \rangle\!\rangle = \langle\!\langle \ln Z \rangle\!\rangle = \lim_{n\to 0}\frac{1}{n}(\langle\!\langle Z^n \rangle\!\rangle - 1) \qquad \text{or, equivalently,} \qquad \langle\!\langle \ln Z \rangle\!\rangle = \lim_{n\to 0}\frac{1}{n}\ln\langle\!\langle Z^n \rangle\!\rangle, \tag{S6.2}$$

with

$$\langle\!\langle Z^n \rangle\!\rangle = \int D\boldsymbol{z}\sum_{\boldsymbol{x}}\exp\left(\sum_u \frac{1}{\gamma}\ln\left[1 + \gamma\beta\left(\frac{NJ_0}{2}\left(\frac{1}{N}\sum_i x_i^u\xi_i\right)^2 - \frac{J_0}{2} + \frac{J}{\sqrt{N}}\sum_{i<j} z_{ij}x_i^u x_j^u\right)\right]_+\right) \tag{S6.3}$$

$$= \frac{1}{(2\pi)^{2n}}\int d\boldsymbol{z}p(\boldsymbol{z})\sum_{\boldsymbol{x}} d\boldsymbol{m}d\hat{\boldsymbol{m}}d\boldsymbol{\mu}d\hat{\boldsymbol{\mu}}\exp\left(\frac{1}{\gamma}\sum_u \ln\left(1 + \gamma\beta\left(\frac{NJ_0}{2}m_u^2 - \frac{J_0}{2} + J\mu_u\right)\right)\right.$$
$$\left. - \sum_u \mathrm{i}\hat{m}_u\left(m_u - \frac{1}{N}\sum_i \xi_i x_i^u\right) - \sum_u \mathrm{i}\hat{\mu}_u\left(\mu_u - \frac{1}{\sqrt{N}}\sum_{i<j} z_{ij}x_i^u x_j^u\right)\right). \tag{S6.4}$$

Recalling that the $\boldsymbol{z}$ couplings are distributed by a centred Gaussian $\mathcal{N}(0,1)$, we can carry out explicit integration of z_{ij}. Noting that $\int D\boldsymbol{z}\, e^{\boldsymbol{\lambda}^\top\boldsymbol{z}} = e^{\boldsymbol{\lambda}^\top\boldsymbol{\lambda}/2}$, the above may be rewritten as,

$$= \frac{1}{(2\pi)^{2n}}\int d\boldsymbol{m}d\hat{\boldsymbol{m}}d\boldsymbol{\mu}d\hat{\boldsymbol{\mu}}\sum_{\boldsymbol{x}}\exp\left(\frac{1}{\gamma}\sum_u \ln\left(1 + \gamma\beta\left(\frac{NJ_0}{2}m_u^2 - \frac{J_0}{2} + J\mu_u\right)\right)\right.$$
$$\left. - \sum_u \mathrm{i}\hat{m}_u\left(m_u - \frac{1}{N}\sum_i \xi_i x_i^u\right) - \sum_u \mathrm{i}\hat{\mu}_u\mu_u + \frac{1}{4N}\sum_{i\neq j}\left(\sum_u \mathrm{i}\hat{\mu}_u x_i^u x_j^u\right)^2\right). \tag{S6.5}$$

The last term in the exponential can be expressed as,

$$N\frac{1}{2}\sum_{u<v}\mathrm{i}\hat{\mu}_u\mathrm{i}\hat{\mu}_v\left(\left(\frac{1}{N}\sum_i x_i^u x_i^v\right)^2 - \frac{1}{N}\right) - N\frac{1}{4}\sum_u (\mathrm{i}\hat{\mu}_u)^2\left(1 - \frac{1}{N}\right). \tag{S6.6}$$

Furthermore, introducing conjugate pair fields for the average of $x_i^u x_i^v$, $\{\boldsymbol{q},\hat{\boldsymbol{q}}\}$ we have,

$$(2\pi)^{-2n-n(n-1)}\int d\boldsymbol{m}d\hat{\boldsymbol{m}}d\boldsymbol{\mu}d\hat{\boldsymbol{\mu}}d\boldsymbol{q}d\hat{\boldsymbol{q}}$$
$$\sum_{\boldsymbol{x}}\exp\left(\frac{1}{\gamma}\sum_u \ln\left(1 + \gamma\beta\left(\frac{NJ_0}{2}m_u^2 - \frac{J_0}{2} + J\mu_u\right)\right) - N\frac{1}{4}\sum_u (\mathrm{i}\hat{\mu}_u)^2\left(1 - \frac{1}{N}\right) - \sum_u \mathrm{i}\hat{\mu}_u\mu_u\right.$$
$$\left. - \sum_u \mathrm{i}\hat{m}_u\left(m_u - \frac{1}{N}\sum_i \xi_i x_i^u\right) - \sum_{u<v}\mathrm{i}\hat{q}_{uv}\left(q_{uv} - \frac{1}{N}\sum_i x_i^u x_i^v\right) + N\frac{1}{2}\sum_{u<v}\mathrm{i}\hat{\mu}_u\mathrm{i}\hat{\mu}_v\left(q_{uv}^2 - \frac{1}{N}\right)\right). \tag{S6.7}$$

Now we can evaluate the integrals by steepest descent

$$= \exp\left\{\frac{1}{\gamma}\sum_u \ln\left(1+\gamma\beta\left(\frac{NJ_0}{2}m_u^2-\frac{J_0}{2}+J\mu_u\right)\right)-N\frac{1}{4}\sum_u(\mathrm{i}\hat{\mu}_u)^2\left(1-\frac{1}{N}\right)\right.$$
$$\left.-\sum_u \mathrm{i}\hat{m}_u m_u-\sum_u \mathrm{i}\hat{\mu}_u\mu_u-\sum_{u<v}\mathrm{i}\hat{q}_{uv}q_{uv}+\frac{1}{2}N\sum_{u<v}\mathrm{i}\hat{\mu}_u\mathrm{i}\hat{\mu}_v\left(q_{uv}^2-\frac{1}{N}\right)+\ln\sum_{\boldsymbol{x}}\exp L\right\}. \tag{S6.8}$$

The overall 2π factor has been left out as we are ultimately concerned with $n \to 0$. With L corresponding to the x_i-dependant part in the argument of the exponential (S5.9). Here, (S6.8) is understood at the saddle-node solution, which, ignoring the $\mathcal{O}(\frac{1}{N})$ terms, corresponds to,

$$\begin{aligned}
\mathrm{i}\hat{m}_u &= \beta'_u N J_0 m_u,\\
\mathrm{i}\hat{\mu}_u &= \beta'_u J,\\
m_u &= \langle x_i^u\rangle,\\
q_{uv} &= \langle x_i^u x_i^v\rangle,\\
i\hat{q}_{uv} &= N\mathrm{i}\hat{\mu}_u\mathrm{i}\hat{\mu}_v q_{uv} = N\left(\beta'_u J\right)^2 q_{uv},\\
\mu_u &= N\frac{1}{2}\sum_v \mathrm{i}\hat{\mu}_v q_{uv}^2 = NJ\frac{1}{2}\sum_v \beta'_v q_{uv}^2,\\
\beta'_u &= \frac{\beta}{1+\gamma\beta\left(N\frac{1}{2}J_0 m_u^2+\mu_u\right)} = \frac{\beta}{1+\gamma\beta N\frac{1}{2}\left(J_0 m_u^2+J^2\sum_v\beta'_v q_{uv}^2\right)}.
\end{aligned} \tag{S6.9}$$

Assuming $q_{uu}=1$, we can rewrite (S6.8) by evaluating at (S6.9). As we are contemplating the $n \to 0$ limit, N is taken large but kept at a fixed value, resulting in

$$\langle\!\langle\varphi_\gamma\rangle\!\rangle = \frac{1}{n}\frac{1}{\gamma}\sum_u \ln\left(1+\gamma\beta\left(\frac{NJ_0}{2}m_u^2-\frac{J_0}{2}+NJ^2\frac{1}{2}\sum_v\beta'_v q_{uv}^2\right)\right)$$
$$-\frac{1}{n}\sum_u \beta'_u N J_0 m_u^2 - N\frac{3}{4n}\sum_{u,v}\beta'_u\beta'_v J^2 q_{uv}^2+\frac{1}{n}\ln\sum_{\boldsymbol{x}}\exp L. \tag{S6.10}$$

In the limit $\gamma \to 0$, we recover the replica free-energy of the SK model

$$\lim_{\gamma\to 0}\langle\!\langle\varphi_\gamma\rangle\!\rangle = -\beta N\frac{J_0}{2n}\sum_u m_u^2 - N\frac{\beta^2 J^2}{4n}\sum_{u,v}q_{uv}^2+\frac{1}{n}\ln\sum_{\boldsymbol{x}}\exp L. \tag{S6.11}$$

1. Replica symmetry

The assumption of replica symmetry implies homogeneous couplings among replicas $q_{uv}=\delta_{uv}+q(1-\delta_{uv})$. Also we will consider $m_u = m$ for the mean field,

$$\langle\!\langle\varphi_\gamma\rangle\!\rangle = \frac{1}{\gamma}\ln\left[1+\gamma\beta\left(\frac{NJ_0}{2}m^2-\frac{J_0}{2}+NJ^2\frac{1}{2}\beta'(1-q^2)\right)\right]_+$$
$$-\beta' N J_0 m^2 - N\frac{3}{4}\beta'^2 J^2(1-q^2)+\frac{1}{n}\ln\sum_{\boldsymbol{x}}\exp L \tag{S6.12}$$

with L carrying the $\boldsymbol{x}$ dependence. We can further simplify evaluating the sum,

$$
\begin{aligned}
\ln \sum_{\boldsymbol{x}} \exp L = & \ln \sum_{\boldsymbol{x}} \exp \left(n\beta' J_0 m \sum_{i,u} \xi_i x_i^u + (\beta' J)^2 q \sum_{i,u<v} x_i^u x_i^v \right) \\
& = \ln \prod_i \sum_{\boldsymbol{x}_i} \exp \left(\sum_u \beta' J_0 m \xi_i x_i^u + \frac{1}{2} \left(\beta' J \sqrt{q} \sum_u x_i^u \right)^2 - \frac{1}{2} n \left(\beta' J\right)^2 q \right) \\
& = \ln \prod_i \int Dz \sum_{\boldsymbol{x}_i} \exp \left(\sum_u \beta' J_0 m x_i^u + \beta' J \sqrt{q} z \sum_u x_i^u - \frac{1}{2} n \left(\beta' J\right)^2 q \right) \\
& = N \ln \int Dz \exp \left(n \ln \left(2\cosh \left(\beta' J_0 m + \beta' J \sqrt{q} z \right) \right) \right) - \frac{1}{2} n N \left(\beta' J\right)^2 q \\
& \approx Nn \int Dz \ln \left(2\cosh \left(\beta' J_0 m + \beta' J \sqrt{q} z \right) \right) - \frac{1}{2} n N \left(\beta' J\right)^2 q. \qquad \text{(S6.13)}
\end{aligned}
$$

We thus obtain

$$
\begin{aligned}
\frac{1}{N} \langle\!\langle \varphi \rangle\!\rangle = & \frac{1}{N\gamma} \ln \left(1 + N\gamma\beta \left(\frac{J_0}{2} m^2 + J^2 \frac{1}{2} \beta' (1-q^2) \right) \right) \\
& - \beta' J_0 m^2 - \frac{3}{4} \beta'^2 J^2 (1-q^2) + \int Dz \ln \left(2\cosh \left(\beta' J_0 m + \beta' J \sqrt{q} z \right) \right) - \frac{1}{2} \left(\beta' J\right)^2 q. \qquad \text{(S6.14)}
\end{aligned}
$$

Extremisation with respect to m and q yields:

$$
\beta' J_0 m = \beta' J_0 \int Dz \tanh \left(\beta' J_0 m + \beta' J \sqrt{q} z \right), \qquad \text{(S6.15)}
$$

$$
\frac{\beta'^2 J^2}{2} (1-q) = \beta' J \frac{1}{2\sqrt{q}} \int Dz \tanh \left(\beta' J_0 m + \beta' J^2 \sqrt{q} z \right) z, \qquad \text{(S6.16)}
$$

leading to the solution, for $\gamma' = N\beta\gamma$:

$$
m = \int Dz \tanh \left(\beta' (J_0 m + J \sqrt{q} z) \right), \qquad \text{(S6.17)}
$$

$$
q = \int Dz \tanh^2 \left(\beta' (J_0 m + J \sqrt{q} z) \right), \qquad \text{(S6.18)}
$$

$$
\beta' = \frac{\beta}{1 + \gamma' \left(\frac{1}{2} J_0 m^2 + \frac{1}{2} \beta' J^2 (1-q^2) \right)}. \qquad \text{(S6.19)}
$$

a. *Critical point*

The solution for $J_0 = 0, J = 1$ at $\gamma' = 0$ has the form

$$
q = \int Dz \tanh^2 \left(\beta J \sqrt{q} z \right). \qquad \text{(S6.20)}
$$

Using a change of variables $\rho = \sqrt{q}$, we can expand around $\rho \to 0$ ($\tanh^2 x = (x - \frac{1}{3}x^2 + \cdots)^2 = x^2 - \frac{2}{3}x^4 + \cdots$ for small x). Noting that $\int Dz\, z^2 = 1$ and $\int Dz\, z^4 = 3$, we obtain

$$
\begin{aligned}
q = & \int Dz \tanh^2 \left(\beta J \rho z \right) = (\beta J)^2 \rho^2 - 2(\beta J)^4 \rho^4 + \mathcal{O}(\rho^6) \\
& = (\beta J)^2 q - 2(\beta J)^4 q^2 + \mathcal{O}(q^3). \qquad \text{(S6.21)}
\end{aligned}
$$

This yields a trivial solution $q = 0$, and a solution

$$
q = \frac{1}{2(\beta J)^4} \left((\beta J)^2 - 1 \right) \qquad \text{(S6.22)}
$$

for $\beta J > 1$, with a slope of

$$\frac{\partial q}{\partial \beta} = \frac{1}{\beta^5 J^4}(2 - (\beta J)^2), \tag{S6.23}$$

which is equal to 1 near the critical point, $\beta J = 1$.

For $\gamma' \neq 0$, we can recover the critical solution by a change of variables $\beta \to \beta'$. For $m = 0$, the solution of (S6.18) for an arbitrary β' is the same as the solution of (S6.20) for $\beta = \beta'$. For each pair of β', q solving (S6.18), we can recover the corresponding inverse temperature from (S6.19) as $\beta = \beta'(1 + \frac{1}{2}\gamma'\beta' J^2(1 - q^2))$.

Following the previous argument, we can show

$$q = \frac{1}{2(\beta' J)^4}((\beta' J)^2 - 1), \tag{S6.24}$$

resulting in that, at the critical β, we must obtain $\beta' J = 1$ and $q = 0$. Then we have

$$\beta' + \beta'^2 J^2 \gamma' \frac{1}{2} = \beta, \tag{S6.25}$$

which, for $\beta' J = 1$, yields the critical inverse temperature

$$\beta_c = J^{-1} + \frac{1}{2}\gamma'. \tag{S6.26}$$

The derivative of β' yields

$$\frac{d\beta'}{d\beta}(1 + \beta'\gamma' J^2) = 1 \tag{S6.27}$$

$$\frac{d\beta'}{d\beta} = \frac{1}{1 + \beta'\gamma' J^2}, \tag{S6.28}$$

resulting in a slope of

$$\frac{\partial q}{\partial \beta} = \frac{\partial q}{\partial \beta'}\frac{\partial \beta'}{\partial \beta} = \frac{1}{\beta'^5 J^4}\frac{2 - (\beta' J)^2}{1 + \beta'\gamma' J^2}, \tag{S6.29}$$

which, for $\beta' = J^{-2}$, diverges at $\gamma' = -1$, resulting in a second-order phase transition.

Supplementary Note 7: Glauber dynamics of dense associative memories

The energy of the dense associative memories (a.k.a., modern Hopfield networks) with a state $\boldsymbol{x} = (x_1, \ldots, x_n) \in \{-1, 1\}^n$ takes the following functional form:

$$\mathcal{F} = -\sum_a F\Big(\sum_i \xi_i^a x_i\Big), \tag{S7.1}$$

where $F(\cdot)$ is a non-linear function. The original formulation of dense associative memories used the rectified polynomial function $F(z) = z^p \cdot \Theta(z)$ with $\Theta(z)$ being the Heaviside step function [20], and other authors have used the exponential function $F(z) = e^z$ [21].

The deterministic update rule of the dense associative memories can be written using the following conditional probability as follows:

$$p(x_k|\boldsymbol{x}_{\setminus k}) = \Theta\big(\Delta\mathcal{F}(\boldsymbol{x})\big), \tag{S7.2}$$

where $\Delta\mathcal{F}(\boldsymbol{x}) = \mathcal{F}(-x_k, \boldsymbol{x}_{\setminus k}) - \mathcal{F}(x_k, \boldsymbol{x}_{\setminus k})$. The energy difference $\Delta\mathcal{F}$ can be expressed as

$$\begin{aligned}\Delta\mathcal{F}(\boldsymbol{x}) &= \sum_a F\Big(\sum_i \xi_i^a x_i\Big) - \sum_a F\Big(-\xi_k^a x_k + \sum_{j\neq k} \xi_j^a x_j\Big) \\ &= \sum_a \bigg(F\Big(\sum_i \xi_i^a x_i\Big) - F\Big(-2\xi_k^a x_k + \sum_i \xi_i^a x_i\Big)\bigg).\end{aligned} \tag{S7.3}$$

Thus, using the shorthand notation $\Delta\epsilon_k^a \coloneqq 2\xi_k^a x_k$ (corresponding to the correlation between the k-th element of the a-th memory and the state) and

$$\Delta F_k^a \coloneqq F\Big(\sum_i \xi_i^a x_i\Big) - F\Big(-\Delta\epsilon_k^a + \sum_i \xi_i^a x_i\Big), \tag{S7.4}$$

the input to the threshold activation function in Eq. (S7.2) can be expressed as

$$\Delta\mathcal{F}(\boldsymbol{x}) = \sum_a \frac{\Delta F_k^a}{\Delta\epsilon_k^a}\Delta\epsilon_k^a = \sum_a w_k^a \Delta\epsilon_k^a, \quad \text{where} \quad w_k^a = \frac{\Delta F_k^a}{\Delta\epsilon_k^a}. \tag{S7.5}$$

Above, we are assuming that $\xi_i^a \neq 0$ so that $\Delta\epsilon_k^a \neq 0$ for simplicity. This implies that, in the dense associative memories, each neuron has distinct effective weights for each memory: the k-th neuron receives an input $\sum_a w_k^a \Delta\epsilon_k^a$, where w_k^a weight $\Delta\epsilon_k^a$, which measures the matching of the state x_k with the memory ξ_k^a. We can also regard the process as the gain modulation of the original weight ξ_k^a attached to the input x_k by w_k^a.

Let us now showcase that this effective weight w_k^a is an increasing function of $\sum_i \xi_i^a x_i$ for $F(z) = z^p \cdot \Theta(z)$ and also for $F(z) = e^z$, and hence it supports the accelerated retrieval of a selected memory.

Let us first consider the case of $F(z) = z^p \cdot \Theta(z)$ with any integer $p \geq 2$. If $\Delta z > 0$, we have

$$\Delta F(z) = F(z) - F(z - \Delta z) = \begin{cases} 0 & z < 0 \\ z^p & 0 \leq z < \Delta z \\ z^p - (z - \Delta z)^p & z \geq \Delta z \end{cases} \tag{S7.6}$$

This function is non-negative for all z. It is also an increasing function of z (for $z \geq \Delta z$, $\partial_z \Delta F(z) = p(z^{p-1} - (z - \Delta z)^{p-1}) > 0$). Similarly, if $\Delta z < 0$, we have

$$\Delta F(z) = \begin{cases} 0 & z < \Delta z \\ -(z - \Delta z)^p & \Delta z \leq z < 0 \\ z^p - (z - \Delta z)^p & z \geq 0 \end{cases} \tag{S7.7}$$

It is a non-positive, decreasing function of z. Hence, $\frac{\Delta F(z)}{\Delta z}$ is a non-negative, increasing function of z. Namely, with $\Delta F(z) = \Delta F_k^a(z)$, $z = \sum_i \xi_i^a x_i$ and $\Delta z = \Delta\epsilon_k^a$, $w_k^a = \frac{\Delta F_k^a}{\Delta\epsilon_k^a}$ is a non-negative, increasing function of $\sum_i \xi_i^a x_i$.

Let us now consider the case of $F(z) = e^z$. In this case we have $\Delta F(z) = e^z - e^{z-\Delta z}$, which is positive if $\Delta z > 0$ and negative if $\Delta z < 0$. The derivative is $\partial_z \Delta F(z) = e^z - e^{z-\Delta z}$, which is positive if $\Delta z > 0$ and negative if $\Delta z < 0$. This guarantees that $\frac{\Delta F_k^a}{\Delta\epsilon_k^a}$ is a positive, increasing function of $\sum_i \xi_i^a x_i$.

The proof can be extended to a differentiable function $F(z)$ if it is increasing $F'(z) > 0$, and convex $F''(z) > 0$. Let $\Delta z > 0$. By the fundamental theorem of calculus, we have

$$\frac{\Delta F(z)}{\Delta z} = \frac{1}{\Delta z}\int_{z-\Delta z}^{z} F'(t)\,\mathrm{d}t. \tag{S7.8}$$

Because $F(z)$ is convex, $F'(z)$ is increasing, so the integral average on the right is an increasing function of z. Positivity follows from $F'(z) > 0$. Similarly for $\Delta z < 0$

$$\frac{\Delta F(z)}{\Delta z} = \frac{1}{\Delta z}\int_{z-\Delta z}^{z} F'(t)\,\mathrm{d}t = \frac{1}{-|\Delta z|}\int_{z+|\Delta z|}^{z} F'(t)\,\mathrm{d}t = \frac{1}{|\Delta z|}\int_{z}^{z+|\Delta z|} F'(t)\,\mathrm{d}t \tag{S7.9}$$

to which the same argument applies. These prove $\frac{\Delta F(z)}{\Delta z}$ is a positive, increasing function of z. We note that the proof can be further extended to non-differentiable convex functions, too.

Therefore, in these systems, as $\sum_i \xi_i^a x_i$ increases (i.e., as the pattern $\boldsymbol{\xi}^a = (\xi_1^a, \ldots, \xi_N^a)$ is retrieved), the effective weights related to a (i.e., w_k^a, $k = 1, \ldots, N$) increase. This accelerates the alignment of x_k with ξ_k^a, ensuring positive feedback. Additionally, retrieval of $\boldsymbol{\xi}^a$ reduces $\sum_i \xi_i^b x_i$ for orthogonal patterns $\boldsymbol{\xi}^b$, lowering their effective weights, thereby suppressing their recall and minimizing interference. This competitive mechanism highlights the superior capacity of these models compared to curved neural networks with uniform temperature scaling. Unlike the effective inverse temperature in the curved networks that depends only on the system's state or energy, the effective weight in updating k-th neuron in the dense associative memories additionally depends on the neuron's state x_k, thus no longer represents a global modulation of neurons.
